# Psychological cost of Hong Kong’s zero-COVID policy: three-wave repeated cross-sectional study of pandemic fatigue, pandemic fear and emotional well-being from peak pandemic to living-with-COVID policy shift

**DOI:** 10.1192/bjo.2025.18

**Published:** 2025-03-24

**Authors:** Sam S. S. Lau, Jason W. L. Fong, Marco C. H. Cheng

**Affiliations:** Research Centre for Environment and Human Health, School of Continuing Education, Hong Kong Baptist University, Hong Kong, China; College of International Education, School of Continuing Education, Hong Kong Baptist University, Hong Kong, China

**Keywords:** COVID-19, mental health, pandemic fatigue, pandemic fear, zero-COVID policy

## Abstract

**Background:**

Hong Kong’s 3-year dynamic zero-COVID policy has caused prolonged exposure to stringent, pervasive anti-epidemic measures, which poses additional stressors on emotional well-being through pandemic fatigue, beyond the incumbent fear of the pandemic.

**Aims:**

To investigate how major policy shifts in the zero-COVID strategy have corresponded with changing relationships between emotional well-being, pandemic fatigue from policy adherence, and pandemic fear, following the pandemic peak to a living-with-COVID policy.

**Method:**

A three-wave repeated cross-sectional study (*N* = 2266) was conducted on the Chinese working-age population (18–64 years) during the peak outbreak (Wave 1), and subsequent policy shifts towards a living-with-COVID policy during the initial relaxation (Wave 2) and full relaxation (Wave 3) of anti-epidemic measures from March 2022 to March 2023. Non-parametric tests, consisting of robust analysis of covariance tests and quantile regression analysis, were performed.

**Results:**

The severity of all measures was lowered after Wave 1; however, extreme pandemic fears reported in Wave 2 (*n* = 38, 7.7%) were associated with worse emotional well-being than the pandemic peak (Wave 1), which then subsided in Wave 3. Pandemic fatigue posed greater negative emotional well-being in Wave 1, whereas pandemic fear was the dominant predictor in Waves 2 and 3.

**Conclusions:**

Pandemic fatigue and pandemic fear together robustly highlight the psychological cost of prolonged pandemic responses, expanding on a framework for monitoring and minimising the unintended mental health ramifications of anti-epidemic policies.

## Background of the zero-COVID policy

Aligning with China’s dynamic zero-COVID policy, Hong Kong has been successful in maintaining a relatively low infection rate since the first case on 23 January 2020, with vastly lower infections during the first 100 days than in mainland China, parts of Europe, Asia and the Middle East.^1^ Bolstered by high public adherence to infection prevention practices instilled by the severe acute respiratory syndrome (SARS) epidemic in 2003, the dynamic zero-COVID policy served as an effective elimination strategy in isolating infections and minimising the risk factors of pandemic outbreaks for the first 2 years in Hong Kong.^2^ The public health and social measures (PHSMs) of the zero-COVID strategy included locating new symptomatic or asymptomatic cases via mass testing, stricter border control and mandatory quarantining of inbound persons and implementing targeted actions against these cases via comprehensive contact tracing and small-scale lockdowns, as well as social distancing measures via mask mandates, work from home arrangements and dining rules. Nevertheless, the early success in pandemic control did not exempt Hong Kong from the global mental health crisis sparked by living under a global pandemic.^3–5^

The immediate and pervasive psychological impacts were of pandemic fears, that is, the fear of adverse effects from infection, or infecting close family members. Challenges in minimising risks for Hong Kong citizens, such as shortages of face masks, inability to work remotely for some and their concern for lax border policies with China, have disrupted daily routines and elevated risk perceptions, leading to a deterioration of mental health.^3,6^ Amid low government trust from major political strife, pandemic fears and self-reliance in managing individual risks were also key motivators for greater adherence to anti-epidemic measures. Pandemic fatigue is an extended public health crisis caused by prolonged exposure to these invasive measures that disturb daily life functions of everyone, regardless of direct experiences with coronavirus.^7^ It is linked with weakening the effectiveness of anti-epidemic measures, which evidently slowed down Hong Kong’s suppression of the third and fourth outbreaks of COVID-19.^2,8^ Because of universal limitations on daily activities for both vaccinated and unvaccinated individuals under the zero-COVID policy, all individuals were at risk of developing pandemic fatigue in Hong Kong.^9^ Individual dispositions of Hong Kong citizens, such as a strong sense of moral responsibility to follow anti-epidemic measures, fostered susceptibility to depression, anxiety and stress symptoms.^10^

Failing to capitalise on the initial success of the dynamic zero-COVID policy, an outbreak of the highly transmissible Omicron variant (BA.2) in January 2022 far exceeded the capabilities of stringent anti-epidemic measures without a highly immunised population.^11^ The healthcare system was overloaded by a surge of cases into the millions, elevating mortality rates of otherwise mild COVID-19 cases without access to basic care.^12^ A sufficient workforce to treat all serious cases, on top of quarantining the huge spike of mild or asymptomatic people, was unfeasible. Mixed messages from government officials further muddled their anti-epidemic response direction by sparking rumours of potential citywide lockdowns and instigating panic-buying behaviours.^13^ Vaccine acceptance was stifled in part because of the high pandemic fatigue, low-risk conditions cultivated under the zero-COVID policy,^14,15^ which remained most tenacious amongst the vulnerable elderly population,^16^ and necessitated an indefinite continuation of PHSMs to reach a population state of herd immunity. On 7 December 2022, China undertook its own unanticipated policy shift out of zero-COVID strategy. This sudden lift in anti-epidemic measures left the healthcare sector and the general population unprepared after extensive stringent policy restrictions to adapt to the sudden, overwhelming surge of infections and deaths from unmitigated exposure to the Omicron variant.^17^

## Research gap and study aim

The early merits of the dynamic zero-COVID policy cannot be discounted in minimising transmissions and deaths. However, the extant literature has seldomly linked mental health outcomes to specific policy shifts themselves, which were common endeavours for countries transitioning out of elimination strategies into living-with-COVID conditions.^18,19^ Worldwide recognition of pandemic fatigue as a risk factor for future outbreaks has prompted analysis of this phenomenon; however, its prevalence has often conflated with its symptoms of reduced motivation to follow anti-epidemic measures. Bottom-up approaches to defining pandemic fatigue have created a dearth in understanding of the roles enacted policies and policymakers have in preventing (not just alleviating) the buildup of pandemic fatigue through better tailored PHSMs. Although emerging demotivation towards preventative measures is generally accepted as the main indicator of pandemic fatigue, it is difficult to differentiate its true cause among individual levels of risk perception, adherence to anti-epidemic measures or other daily life disruptions.^20^ This study aimed to gauge the strength of effect from pandemic fatigue and pandemic fear as predictors of emotional well-being in the Hong Kong Chinese population, and to explore how this relationship differs across multiple key policy shifts of the dynamic zero-COVID policy from the first major outbreak of the Omicron variant to the end of COVID-19 as a public health emergency.

## Methods

### Study design and participants

**Fig. 1:**
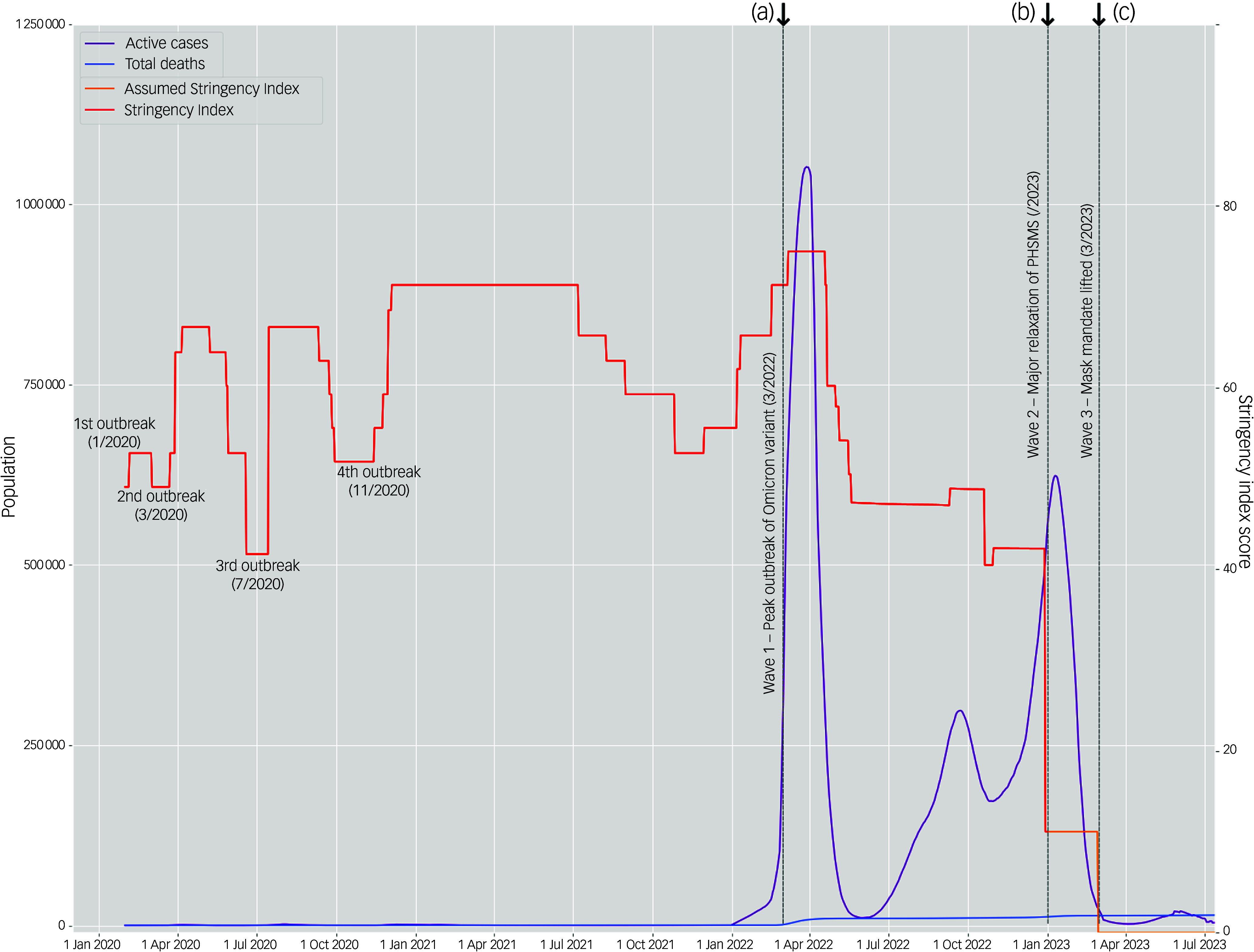
Timeline of the case statistics, outbreaks and policy responses during the COVID-19 pandemic in Hong Kong from 1 January 2020 to July 2023. *Note:* the time frames designated in our study are annotated as (a) Wave 1, (b) Wave 2 and (c) Wave 3, investigating the period during and after the first belated outbreak in Hong Kong’s pandemic timeline. Active cases and total deaths data collated from the Centre of Health Protection, supplemented by Worldometer.^21,22^ Data on the Stringency Index, a composite measure of the strictness of nine possible types of anti-epidemic policy responses issued by the government, was sourced from the Oxford Coronavirus Government Response Tracker (OxGRT) project but does not extend past 31 December 2022.^23^ The Assumed Stringency Index continues the Stringency Index line past the available data based on the successive pandemic developments. See further comparisons with other countries in Supplementary Appendix 1. PHSMs, public health and social measures.

This study was part of a larger study titled ‘COVID-19 pandemic and health’. We adopted a repeated, cross-sectional, questionnaire-based design to gather data from three time points of significant policy shifts, illustrated in Fig. 1, between March 2022 and March 2023.

- Peak outbreak wave (Wave 1) surveyed from 7 March to 23 April 2022, during which the Omicron (BA.2) outbreak exceeded a million active cases with a moving average of 21 685 daily cases reported in the first week from 7 to 13 March 2022.^21^ The escalation of policy stringency during this period corresponded with the launch of the Vaccine Pass arrangement on 24 February 2022, a mandatory contract tracing mobile application for granting access to public premises for vaccinated citizens, while gathering bans and compulsory COVID-19 testing were still in effect for all citizens.
- Initial relaxation wave (Wave 2) surveyed from 3 to 21 January 2023, proceeded PHSM relaxations during the height of the second Omicron (BA.5) wave with a moving average of 2600 daily cases reported in the first week from 3 to 9 January 2023.^21^ Vaccine Pass requirements, mandatory quarantine and testing for non-vulnerable populations and social distancing measures, barring the mask mandate, were scrapped on 29 December 2022.
- Full relaxation wave (Wave 3) surveyed from 1 to 10 March 2023, where COVID-19 cases have substantially subsided, given the moving average of 50 daily cases reported in the first week from 1 to 7 March 2023.^22^ This wave aimed to survey initial responses following the 945-day long mask mandate being lifted on the same day from 1 March 2023 onwards.

Online questionnaires in Chinese were hosted on the QuestionPro platform and disseminated via social media (i.e. Facebook, WhatsApp and WeChat) and emails, based on convenience and snowball sampling at each wave. Written informed consent was obtained from all participants before data collection. The inclusion criteria included those who were living in Hong Kong, aged 18 or above, could read and understand Chinese and had no COVID-19 infections within 1 week. The authors assert that all procedures contributing to this work comply with the ethical standards of the relevant national and institutional committees on human experimentation and with the Helsinki Declaration of 1975, as revised in 2013. All procedures involving human participants/patients were approved by the research ethics committee of Hong Kong Baptist University (REC/21-22/0353, REC/22-23/0336). In the report writing of this observational study, we followed the Strengthening the Reporting of Observational Studies in Epidemiology (STROBE) statement.^24^

We received a total of 3587 responses, comprising 1580 (Wave 1), 1078 (Wave 2) and 929 (Wave 3) group responses. After filtering for incomplete responses, excluding based on the lie scale and omitting respondents past Hong Kong’s retirement age of 65 because of low case representation in each wave (0.8–3.8%), we retained a valid sample of 1132 (71.6%), 493 (45.7%) and 641 (69.0%), respectively, for data analysis. Table 1 presents the sample characteristics. Some 70.8%, 64.9% and 55.9%, respectively, of respondents were female between our three waves. Our Wave 2 sample was younger with a mean age (27.02 ± 11.32), compared to Waves 1 (33.58 ± 12.15) and 3 (34.23 ± 13.95). Correspondingly, 71.9%, 44.4% and 56.8% were engaged in work arrangements between our three waves, and 21.2%, 51.3% and 31.2% were students, respectively. Hong Kong’s outbreaks and vaccination trends were represented in our samples, such as the large uptick in COVID-19 infections observed from Wave 1 (22.7%) to Wave 2 (56.6%), while vaccination uptake at three doses rose between Wave 1 (34.3%) and Wave 2 (82.6%). Beliefs in herd immunity were initially in agreement of its effectiveness in Wave 1 (66.3%), but this rescinded towards neutral responses, almost doubling from Wave 1 (18.3%) to Wave 2 (32.7%) and Wave 3 (32.1%).

**Table 1:** Distribution of demographic variables, COVID-19 status and beliefs and scale scores between waves (*N* = 2266)

Variables	Wave 1 (*n*_1_ = 1132)	Wave 2 (*n*_2_ = 493)	Wave 3 (*n*_3_ = 641)	*χ* ^2^
	mean (s.d.) *n* (%)	mean (s.d.) *n* (%)	mean (s.d.) *n* (%)	
(a) Demographic variables				
Gender				
Male	331 (29.2%)	173 (35.1%)	283 (44.1%)	40.17***
Female	801 (70.8%)	320 (64.9%)	358 (55.9%)	
Age	**33.58 (12.15)**	**27.02 (11.32)**	**34.23 (13.95)**	
18–24	350 (30.9%)	306 (62.1%)	242 (37.8%)	152.24***
25–44	507 (44.8%)	121 (24.5%)	220 (34.3%)	
45–64	275 (24.3%)	66 (13.4%)	179 (27.9%)	
Employment status				
Full-time workers	673 (59.5%)	177 (35.9%)	300 (46.8%)	
Others (part-time/freelance/temporary work)	141 (12.5%)	42 (8.5%)	64 (10.0%)	
Unemployed	17 (1.5%)	4 (0.8%)	12 (1.9%)	
Retired	15 (1.3%)	5 (1.0%)	25 (3.9%)	
Students	240 (21.2%)	253 (51.3%)	200 (31.2%)	
Homemakers	46 (4.1%)	12 (2.4%)	40 (6.2%)	
(b) COVID-19 status and beliefs				
Been infected by COVID-19 before?				
No (0)	875 (77.3%)	206 (41.8%)	188 (29.3%)	429.82***
Yes (1)	257 (22.7%)	279 (56.6%)	453 (70.7%)	
Undisclosed	–	8 (1.6%)	–	
No. of vaccination(s)	**2.23 (0.68)**	**2.76 (0.60)**	**2.72 (0.62)**	
0 dose (0)	35 (3.1%)	10 (2.0%)	16 (2.5%)	514.00***
1 dose (1)	55 (4.9%)	14 (2.8%)	11 (1.7%)	
2 doses (2)	654 (57.8%)	62 (12.6%)	107 (16.7%)	
3 doses or above (3)	388 (34.3%)	407 (82.6%)	525 (78.8%)	
Fully vaccinated				
Not enough (3 below) (0)	744 (65.7%)	86 (17.4%)	134 (20.9%)	498.70***
Enough (3 or above) (1)	388 (34.3%)	407 (82.6%)	507 (79.1%)	
Do you agree that herd immunity is effective?	**3.86 (1.20)**	**3.40 (0.94)**	**3.54 (0.91)**	
Strongly disagree (1)	59 (5.2%)	12 (2.4%)	11 (1.7%)	271.95***
Disagree (2)	116 (10.2%)	56 (11.4%)	68 (10.6%)	
Neutral (3)	207 (18.3%)	161 (32.7%)	206 (32.1%)	
Agree (4)	298 (26.3%)	161 (32.7%)	274 (42.7%)	
Strongly agree (5)	452 (39.9%)	47 (9.5%)	82 (12.3%)	
I don’t know	–	56 (11.4%)	–	
(c) Scale scores				
COVID-19 Burnout Frequency Scale (CV-19 BFS)	**20.03 (8.91)**	**15.24 (7.59)**	**15.27 (8.25)**	
Low burnout (5–12)	292 (25.8%)	228 (46.2%)	293 (45.7%)	141.81***
Medium burnout (13–25)	487 (43.0%)	196 (39.8%)	261 (40.7%)	
High burnout (26–35)	353 (31.2%)	69 (14.0%)	87 (13.6%)	
Fear of COVID-19 Scale (FCV-19S)	**16.41 (5.54)**	**10.71 (4.34)**	**10.77 (4.82)**	
Low fear (<17.5)	678 (59.9%)	455 (92.3%)	584 (91.1%)	310.87***
Extreme fear (>17.5)	454 (40.1%)	38 (7.7%)	57 (8.9%)	
Depression, Anxiety and Stress Scale-21 (DASS-21)	**29.89 (23.95)**	**22.95 (23.94)**	**19.65 (21.80)**	
Depression	**10.11 (9.15)**	**7.52 (8.71)**	**6.05 (7.61)**	
Normal (0–9)	603 (53.3%)	323 (65.5%)	473 (73.8%)	78.41***
Mild (10–13)	170 (15.0%)	58 (11.8%)	59 (9.2%)	
Moderate (14–20)	216 (19.1%)	68 (13.8%)	71 (11.1%)	
Severe (21–27)	74 (6.5%)	24 (4.9%)	22 (3.4%)	
Extremely severe (28–42)	69 (6.1%)	20 (4.1%)	16 (2.5%)	
Anxiety	**7.79 (7.44)**	**7.08 (7.71)**	**5.96 (7.00)**	
Normal (0–7)	640 (56.5%)	297 (60.2%)	435 (67.9%)	25.04**
Mild (8–9)	103 (9.1%)	47 (9.5%)	43 (6.7%)	
Moderate (10–14)	218 (19.3%)	77 (15.6%)	97 (15.1%)	
Severe (15–19)	73 (6.4%)	28 (5.7%)	29 (4.5%)	
Extremely severe (20–42)	98 (8.7%)	44 (8.9%)	37 (5.8%)	
Stress	**12.09 (9.29)**	**8.61 (8.87)**	**7.83 (8.43)**	
Normal (0–14)	760 (67.1%)	398 (80.7%)	526 (82.1%)	69.78***
Mild (15–18)	118 (10.4%)	36 (7.3%)	51 (8.0%)	
Moderate (19–25)	141 (12.5%)	28 (5.7%)	30 (4.7%)	
Severe (26–33)	77 (6.8%)	21 (4.3%)	26 (4.1%)	
Extremely severe (34–42)	36 (3.2%)	10 (2.0%)	8 (1.2%)	

*Note*: Bold statistics denote the mean and s.d. of the variable in its corresponding wave. *** *P* < 0.001; ** *P* < 0.01; * *P* < 0.05.

### Instruments

#### Demographics and COVID-19 status and beliefs

Surveys contained self-reported demographic questions and COVID-19 questions on vaccination statuses to gauge population levels of vaccine hesitancy, whether participants have contracted COVID-19 before and their confidence in the effectiveness of herd immunity – the purported goal of the dynamic zero-COVID policy.

#### COVID-19 Burnout Frequency Scale

The COVID-19 Burnout Frequency Scale (CV-19 BFS) was used. This validated 5-item scale, which measures pandemic fatigue in relation to the COVID-19 pandemic, has excellent internal consistency (Cronbach’s *α* = 0.90).^25^ The scale focuses on the frequency of burnout in coping with COVID-19 anti-epidemic measures through symptoms of emotional exhaustion and physical tiredness, boredom from travel restrictions or a loss of control in life, as well as stress, confusion, hopelessness and frustration in adherence with prevention measures. Items were rated on a 7-point frequency-based Likert scale: never (1); a few times a year (2); at least once a month (3); several times a month (4); once a week (5); several times a week (6); and once per day or more (7). Discriminative item analysis was performed on the original data-set to validate the sensitivity of each item in distinguishing discrete levels of pandemic fatigue.^25^ High and low subgroup criterion scores were derived from the discrimination index of the top 27% and bottom 27% of CV-19 BFS total score distributions, which yielded good-to-excellent discrimination indices per item (low burnout: 5–12; medium burnout: 13–25; high burnout: 26–35).^26^

#### Fear of COVID-19 Scale

The Fear of COVID-19 Scale (FCV-19S) was used to measure the level of pandemic fear for COVID-19. The scale showed good internal consistency (Cronbach’s *α* = 0.82) and moderate test–retest reliability, as assessed by the intraclass correlation coefficient (ICC = 0.72).^27^ Participants indicated their level of agreement with the statements on a 5-point Likert scale from strongly disagree (1) to strongly agree (5) for a total score between 7 and 35, with higher scores indicating greater pandemic fears. Past studies demonstrate the cross-cultural applicability of the scale.^28–30^ An upscaled cut-off point of 17.5 or above has been recommended to indicate extreme fear of COVID-19 within Chinese populations.^30^

#### Depression Anxiety Stress Scale-21

The Depression Anxiety Stress Scale-21 (DASS-21) was used to assess emotional well-being through the states of depression, anxiety and psychological stress symptoms.^31,32^ The scale has demonstrated good internal consistency across its three subscales of depression (Cronbach’s *α* = 0.94), anxiety (Cronbach’s *α* = 0.87) and stress (Cronbach’s *α* = 0.91).^31^ It has been validated for clinical and non-clinical samples.^33,34^ We used the validated Chinese version.^35^ Participants were asked to rate the extent to which they have experienced each state over the past week on a 4-point frequency Likert scale from never (0) to almost always (3) for total scores of 0–21 (0–42 adjusted) per subscale; higher scores indicate greater negative emotional well-being outcomes. The scoring distinguishes between five levels of depression, anxiety and stress symptoms: normal, mild, moderate, severe and extremely severe.^32^

The internal consistency of the above instruments were acceptable at all three waves (Supplementary Appendix 2 for McDonald’s omega and Cronbach’s alpha values).

### Statistical analysis

Data analysis was conducted using IBM SPSS Statistics for Windows, Version 29.0 (IBM Corporation, Armonk, New York) and the software R for Windows, Version 4.4.1 (R Foundation for Statistical Computing, Vienna, Austria; see https://www.R-project.org/). Statistical tests utilised a two-tailed test for *P*-values < 0.05 to be considered significant. Non-parametric tests were chosen to analyse the non-normal distributions of emotional well-being (DASS-21) scores because of floor effects (see Supplementary Appendix 3). Robust analysis of covariance (ANCOVA) tests were performed with the WRS package in R to probe for significant differences in 20% trimmed mean scores of pandemic fatigue (CV-19 BFS), pandemic fear (FCV-19S) and emotional well-being outcomes between waves,^36^ controlling for sizeable group differences in gender and age as covariates. Non-normal distributions were treated with iterated analysis of variance (ANOVA) tests across subgroups representing population demographics (gender by age group) for weighted *F*-scores and *P*-values calculated using 20% trimmed means, which is the mean after omitting 20% of scores from both tail ends of the distribution for a measure of central tendency less sensitive to outliers or heavy-tailed distributions, while controlling for the probability of Type I errors.^36^ In addition, categorical levels of pandemic fatigue (low burnout, medium burnout, high burnout) and pandemic fear (extreme fear of COVID-19, low fear of COVID-19) were plotted between waves as five individual groups for ANCOVA tests of 20% trimmed mean scores in emotional well-being. Spearman’s *R* was used to explore the relationship between all scales, and a bootstrapped test for multicollinearity using the MTest package in R examined their appropriateness for regression analyses.^37^ Spearman’s *R* was used to explore the relationship between all scales, and a bootstrapped test for multicollinearity using the MTest package in R examined their appropriateness for regression analyses.^37^ Quantile regression analysis was conducted to examine for heterogenous relationships between the main effects of pandemic fatigue and pandemic fear on predicted emotional well-being scores, where gender, age, COVID-19 status and beliefs were also predictors. Under multiple conditional quantiles at 25%, 50% and 75%, the differences in strength of association of predictors can be measured at the median (50%) or higher (75%) and lower (25%) prevalences of depression, anxiety and stress symptoms.

## Results

### Estimating significant wave differences in scale scores

A robust ANCOVA between our three waves indicated highly significant differences in male and female, 18–24-year-old respondents’ scores for CV-19 BFS (male: *F*_(2,316)_ = 22.73, female: *F*_(2,579)_ = 70.48; *P* < 0.001), FCV-19S (male: *F*_(2,316)_ = 44.41, female: *F*_(2,579)_ = 121.14; *P* < 0.001), depression (male: *F*_(2,316)_ = 4.04, *P* = 0.019; female: *F*_(2,579)_ = 43.86, *P* < 0.001) and stress (male: *F*_(2,316)_ = 5.23, *P* = 0.007; female: *F*_(2,579)_ = 24.75, *P* < 0.001), which trended towards declining post-Wave 1 (see Table 2). Anxiety was the most pervasive symptom of negative emotional well-being, exhibited by over a quarter of respondents across all waves at a moderate or higher level, and was not alleviated for 18–24-year-old male respondents (*F*_(2,316)_ = 1.32, *P* = 0.272), unlike for female respondents (*F*_(2,579)_ = 17.45, *P* < 0.001). Overall, older respondents aged 45–64 reported the least sensitivity to changes in emotional well-being outcomes between policy shifts at different waves, such as in depression for female respondents (*F*_(2,316)_ = 3.00, *P* = 0.057) and anxiety for both genders (male: *F*_(2,316)_ = 1.06, *P* = 0.356; female: *F*_(2,579)_ = 0.13, *P* < 0.875). Notably, pandemic fatigue was also invariant in this age group between waves (*F*_(2,316)_ = 0.96, *P* = 0.392).

**Table 2:** Differences in pandemic fatigue, pandemic fear and emotional well-being between waves, controlling for gender and age (*N* = 2266)

Measures	*F*	*P*	Pairwise comparisons
CV-19 BFS	Weighted *F* = **30.79**	<0.001–0.392	Wave 1 > Wave 2; Wave 1 > Wave 3
Male/18–24	**22.73**	<0.001	
Male/25–44	**12.63**	<0.001	
Male/45–64	0.96	0.392	
Female/18–24	**70.48**	<0.001	
Female/25–44	**28.10**	<0.001	
Female/45–64	**5.71**	0.005	
FCV-19S	Weighted *F* = **77.00**	<0.001	Wave 1 > Wave 2; Wave 1 > Wave 3
Male/18–24	**44.41**	<0.001	
Male/25–44	**36.67**	<0.001	
Male/45–64	**36.98**	<0.001	
Female/18–24	**121.14**	<0.001	
Female/25–44	**84.33**	<0.001	
Female/45–64	**75.18**	<0.001	
DASS-21 – depression	Weighted *F* = **18.76**	<0.001–0.057	Wave 1 > Wave 3
Male/18–24	**4.04**	0.020	
Male/25–44	**12.67**	<0.001	
Male/45–64	**8.44**	<0.001	
Female/18–24	**43.86**	<0.001	
Female/25–44	**16.82**	<0.001	
Female/45–64	3.00	0.057	
DASS-21 – anxiety	Weighted *F* = 6.03	<0.001–0.875	No significant differences
Male/18–24	1.32	0.272	
Male/25–44	**4.24**	0.018	
Male/45–64	1.06	0.356	
Female/18–24	**17.45**	<0.001	
Female/25–44	3.03	0.053	
Female/45–64	0.13	0.875	
DASS-21 – stress	Weighted *F* **= 12.78**	<0.001–0.012	Wave 1 > Wave 3
Male/18–24	**5.23**	0.007	
Male/25–44	**12.48**	<0.001	
Male/45–64	**4.89**	0.012	
Female/18–24	**24.75**	<0.001	
Female/25–44	**12.20**	<0.001	
Female/45–64	**4.92**	0.010	

CV-19 BFS, COVID-19 Burnout Frequency Scale; FCV-19S, Fear of COVID-19 Scale; DASS-21, Depression Anxiety Stress Scale-21.

*Note:* Multiple comparisons were given by 2267 design points weighted by pairs of gender (two groups) and age groups (three groups) scaled to the population sample and using the 20% trimmed means of scale scores between waves. Significant *F*-scores are displayed in bold. Pairwise comparisons indicate a significant global hypothesis of different distributions at a 5% significance level when controlling for the effects of gender and age.

**Fig. 2:**
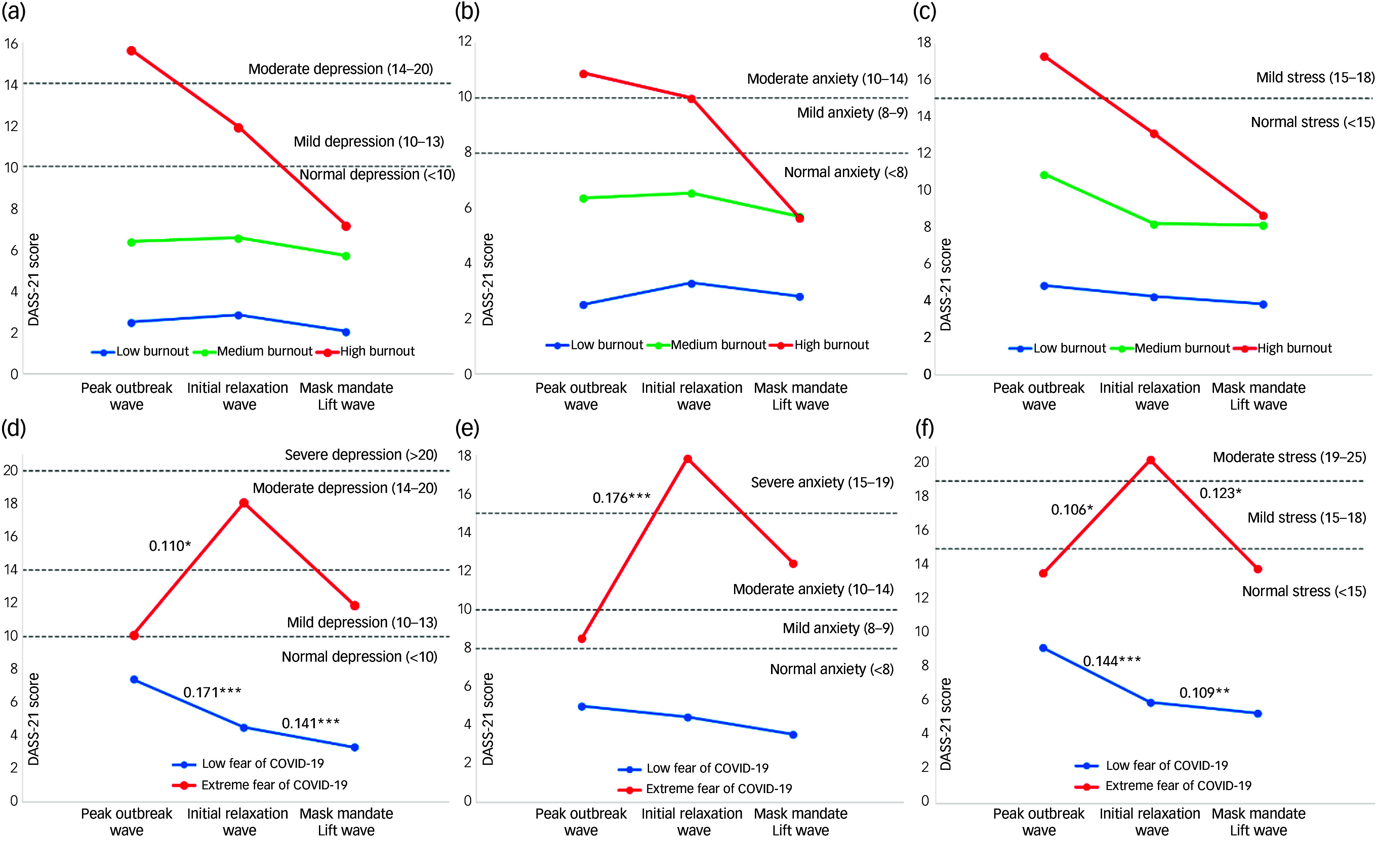
Comparison of 20% trimmed mean scores of depression, anxiety and stress in three waves by pandemic fatigue (a)–(c) and pandemic fear (d)–(f) cut-off scores. *Note:* low, medium and high burnout, indicating levels of pandemic fatigue severity, denote respondents with a COVID-19 Burnout Frequency Scale score between 5 and 12, 13 and 25 and 26 and 35, respectively. Extreme fear of COVID-19 and low fear of COVID-19 denote respondents with a Fear of COVID-19 Scale score above 17.5 and below 17.5, respectively. Pairwise comparisons between waves were given by a robust analysis of covariance using 20% trimmed means on four groups split by gender and the median of population ages. Effect sizes were presented as the explanatory measure of effect size (ξ^2^).^38^ Averaged effect sizes (ξ^2^) were annotated for pairwise comparisons with the lowest groups’ significance levels. Small effect size: ξ^2^ = 0.14; medium effect size: ξ^2^ = 0.34; large effect size: ξ^2^ = 0.52. *** *P* > 0.001; ** *P* > 0.01; * *P* > 0.05. DASS-21, Depression Anxiety Stress Scale-21.

As shown earlier in Table 1, the proportion of Wave 1 respondents experiencing high burnout (31.2%) and extreme fears of COVID-19 (40.1%) plateaued in the subsequent waves, where the percentage of respondents under high burnout (Wave 2: 14.0%; Wave 3: 13.6%) and extreme fears of COVID-19 (Wave 2: 7.7%; Wave 3: 8.9%) remained relatively stable. However, after factoring for how populations under different pandemic fatigue and pandemic fear severity levels correlated with emotional well-being (see Supplementary Appendix 4), this unveiled large differences in 20% trimmed means for emotional well-being contingent on extreme fear of COVID-19 symptoms in Wave 2. As illustrated in Fig. 2, Wave 2 respondents under extreme pandemic fear (*n* = 38; 7.7%) exhibited greater negative emotional well-being outcomes than Wave 1 respondents (depression: = 8.32, *P* = 0.003–0.013; anxiety: = 9.69, *P* < 0.001; stress: = 7.28, *P* = 0.010–0.038) and higher stress symptoms than Wave 3 respondents = 8.20, *P* = 0.022–0.027), whilst controlling for gender and age (Supplementary Appendix 5). The declines in negative emotional well-being for respondents with high, medium and low levels of pandemic fatigue between waves were not all significant between gender and age groups, while pandemic fear produced more uniform effects on emotional well-being across our sample population.

### Predictors of emotional well-being

Spearman’s rank correlations were significant between scale measures at every wave and all predictor variables were assessed for multicollinearity (see Supplementary Appendix 6), which was only detected among age, employment and student enrolment. Age was retained as the more informative demographic factor in our quantile regression analysis (Table 3). Prediction models were assessed for their goodness-of-fit as *R* ^1^ by Koenker and Machado,^39^ a pseudo *R* ^2^ between 0 and 1 attributable as a local measure of each individual quantile based on minimising the sum of weighted deviations.

**Table 3:** Predicting emotional well-being in Waves 1–3 at the 25%, 50% and 75% quantiles using pandemic fatigue and pandemic fear, and other factors

Wave 1: peak outbreak wave (*n* = 1132)									
Independent variables	Depression			Anxiety			Stress		
	25% *B* (s.e.)	50% *B* (s.e.)	75% *B* (s.e.)	25% *B* (s.e.)	50% *B* (s.e.)	75% *B* (s.e.)	25% *B* (s.e.)	50% *B* (s.e.)	75% *B* (s.e.)
Intercept	−5.00 (1.27)***	−1.48 (1.62)	4.21 (2.12)*	−4.20 (1.17)***	−2.88 (1.39)*	0.26 (2.12)	−0.86 (1.68)	−5.11 (1.91)	1.22 (2.62)
Gender (0 = male)	−0.07 (0.38)	−0.42 (0.49)	−3.09 (0.63)***	0.24 (0.35)	0.09 (0.42)	−0.65 (0.64)	−0.04 (0.51)	−0.02 (0.02)	−0.05 (0.03)
Age	−0.03 (0.02)	−0.06 (0.02)**	−0.06 (0.26)*	−0.01 (0.01)	−0.06 (0.02)***	−0.07 (0.03)**	−0.01 (0.02)	−0.02 (0.02)	−0.05 (0.32)
No. of vaccinations taken (0–3+)	0.04 (2.69)	−0.13 (3.44)	−1.05 (0.45)*	−0.30 (0.25)	−0.08 (0.30)	−0.45 (0.45)	0.09 (0.36)	−0.08 (0.41)	−0.39 (0.56)
Contracted COVID-19 before (0 = have not)	0.67 (0.42)	0.41 (0.54)	0.57 (0.70)	1.72 (0.39)***	1.35 (0.46)**	0.77 (0.70)	0.75 (0.56)	0.97 (0.63)	−0.20 (0.87)
Herd immunity outlook (1–5)	−0.28 (0.16)	−0.24 (0.21)	−0.31 (0.27)	−0.03 (0.15)	0.07 (0.18)	−0.26 (0.27)	0.34 (0.21)	−0.25 (0.25)	−0.21 (0.33)
CV-19 BFS	0.33 (0.02)***	0.52 (0.03)***	0.70 (0.04)***	0.17 (0.02)***	0.28 (0.02)***	0.45 (0.04)***	0.35 (0.03)***	0.54 (0.03)***	0.63 (0.04)***
FCV-19S	0.25 (0.03)***	0.24 (0.04)***	0.25 (0.05)***	0.28 (0.03)***	0.33 (0.04)***	0.39 (0.05)***	0.42 (0.04)***	0.42 (0.05)***	0.44 (0.07)***
Pseudo *R* ^2^	0.154	0.222	0.257	0.099	0.170	0.207	0.171	0.209	0.221
CV-19 BFS Δ*R* ^2^	0.077	0.119	0.154	0.031	0.065	0.094	0.075	0.107	0.128
FCV-19S Δ*R* ^2^	0.017	0.013	0.012	0.033	0.041	0.033	0.040	0.036	0.023

Wave 2: initial relaxation wave (*n* = 493)									
Independent variables	Depression			Anxiety			Stress		
	25% *B* (s.e.)	50% *B* (s.e.)	75% *B* (s.e.)	25% *B* (s.e.)	50% *B* (s.e.)	75% *B* (s.e.)	25% *B* (s.e.)	50% *B* (s.e.)	75% *B* (s.e.)
Intercept	−3.35 (1.17)**	−0.89 (2.55)	0.56 (4.27)	−5.05 (0.98)***	−5.33 (2.15)*	−3.40 (3.42)	−6.68 (1.58)***	−2.24 (2.64)	0.256 (3.95)
Gender (0 = male)	−0.05 (0.34)	−0.12 (0.75)	−0.35 (1.26)	0.10 (2.90)	0.23 (0.63)	0.41 (1.01)	0.22 (0.47)	0.43 (0.78)	0.48 (1.17)
Age	−0.06 (0.01)***	−0.11 (0.03)**	−0.14 (0.05)**	−0.03 (0.01)*	−0.08 (0.03)**	−0.07 (0.04)	−0.05 (0.02)*	−0.09 (0.03)**	−0.08 (0.05)
No. of vaccinations taken (0–3+)	−0.27 (0.26)	−1.05 (0.57)	−1.18 (0.95)	−0.12 (0.22)	−0.25 (0.48)	−1.00 (0.76)	0.17 (0.35)	−0.80 (0.59)	−1.51 (0.88)
Contracted COVID-19 before (0 = have not)	0.04 (0.33)	−0.16 (0.73)	−0.65 (1.22)	0.04 (0.28)	−0.11 (0.61)	0.64 (0.97)	0.25 (0.45)	0.09 (0.75)	−0.36 (1.13)
Herd immunity outlook (1–5)	0.11 (0.17)	−0.26 (0.38)	0.09 (0.64)	0.04 (0.15)	−0.05 (0.32)	0.06 (0.51)	0.01 (0.22)	−0.45 (0.40)	−0.07 (0.60)
CV-19 BFS	0.08 (0.02)***	0.26 (0.05)***	0.38 (0.08)***	0.04 (0.02)*	0.16 (0.04)***	0.25 (0.07)***	0.08 (0.03)**	0.21 (0.05)***	0.38 (0.08)***
FCV-19S	0.59 (0.04)***	0.90 (0.08)***	1.12 (0.14)***	0.76 (0.03)***	1.09 (0.07)***	1.25 (0.11)***	0.92 (0.05)***	1.13 (0.09)***	1.18 (0.13)***
Pseudo *R* ^2^	0.101	0.232	0.246	0.159	0.241	0.284	0.161	0.243	0.235
CV-19 BFS Δ*R* ^2^	0.008	0.028	0.036	0.004	0.015	0.018	0.007	0.019	0.040
FCV-19S Δ*R* ^2^	0.093	0.113	0.119	0.135	0.178	0.182	0.128	0.155	0.127

Wave 3: full relaxation wave (*n* = 641)									
Independent variables	Depression			Anxiety			Stress		
	25% *B* (s.e.)	50% *B* (s.e.)	75% *B* (s.e.)	25% *B* (s.e.)	50% *B* (s.e.)	75% *B* (s.e.)	25% *B* (s.e.)	50% *B* (s.e.)	75% *B* (s.e.)
Intercept	−1.75 (0.29)***	0.41 (2.02)	1.94 (2.57)	−2.80 (0.46)***	−1.09 (1.67)	3.63 (2.79)	−0.35 (0.86)***	−3.09 (2.44)	8.81 (3.64)*
Gender (0 = male)	−0.00 (0.08)	−1.13 (0.53)*	−1.27 (0.67)	−0.00 (0.12)	−0.42 (0.44)	−0.00 (0.73)	−0.00 (0.23)	−0.17 (0.64)	−0.34 (0.95)
Age	−0.00 (0.00)	−0.07 (0.02)***	−0.09 (0.02)***	−0.00 (0.00)	−0.04 (0.02)*	−0.08 (0.03)**	−0.00 (0.01)	−0.05 (0.02)*	−0.07 (0.03)*
No. of vaccinations taken(0–3+)	−0.00 (0.06)	−0.01 (0.43)	−0.50 (0.55)	−0.00 (0.10)	−0.49 (0.36)	−0.58 (0.60)	−0.00 (0.19)	0.05 (0.52)	−0.93 (0.78)
Contracted COVID-19 before (0 = have not)	−0.00 (0.08)	−0.91 (0.58)	−0.50 (0.55)	−0.00 (0.13)	−0.09 (0.48)	−1.63 (0.80)	0.00 (0.25)	−0.35 (0.70)	−1.84 (1.05)
Herd immunity outlook (1–5)	−0.00 (0.04)	−0.35 (0.30)	−0.45 (0.38)	−0.00 (0.07)	−0.39 (0.25)	−0.85 (0.41)*	−0.00 (0.13)	−0.18 (0.36)	−0.75 (0.53)
CV-19 BFS	0.00 (0.00)	0.16 (0.03)***	0.41 (0.04)***	0.00 (0.01)	0.09 (0.03)**	0.26 (0.05)***	0.00 (0.01)	0.20 (0.04)***	0.35 (0.06)***
FCV-19S	0.25 (0.01)***	0.60 (0.06)***	0.81 (0.07)***	0.40 (0.01)***	0.83 (0.05)***	0.91 (0.08)***	0.50 (0.02)***	0.84 (0.07)***	0.63 (0.10)***
Pseudo *R* ^2^	0.028	0.140	0.227	0.059	0.154	0.235	0.065	0.147	0.176
CV-19 BFS Δ*R* ^2^	0.000	0.013	0.045	0.000	0.010	0.024	0.000	0.018	0.042
FCV-19S Δ*R* ^2^	0.028	0.070	0.090	0.056	0.123	0.132	0.065	0.099	0.057

CV-19 BFS, COVID-19 Burnout Frequency Scale; FCV-19S, Fear of COVID-19 Scale.

*Note:* Pseudo *R* ^2^ was calculated using Koenker and Machado’s goodness-of-fit measure (*R* ^1^)^39^ for individual quantiles.

*** *P* < 0.001; ** *P* < 0.01; * *P* < 0.05.

**Fig. 3:**
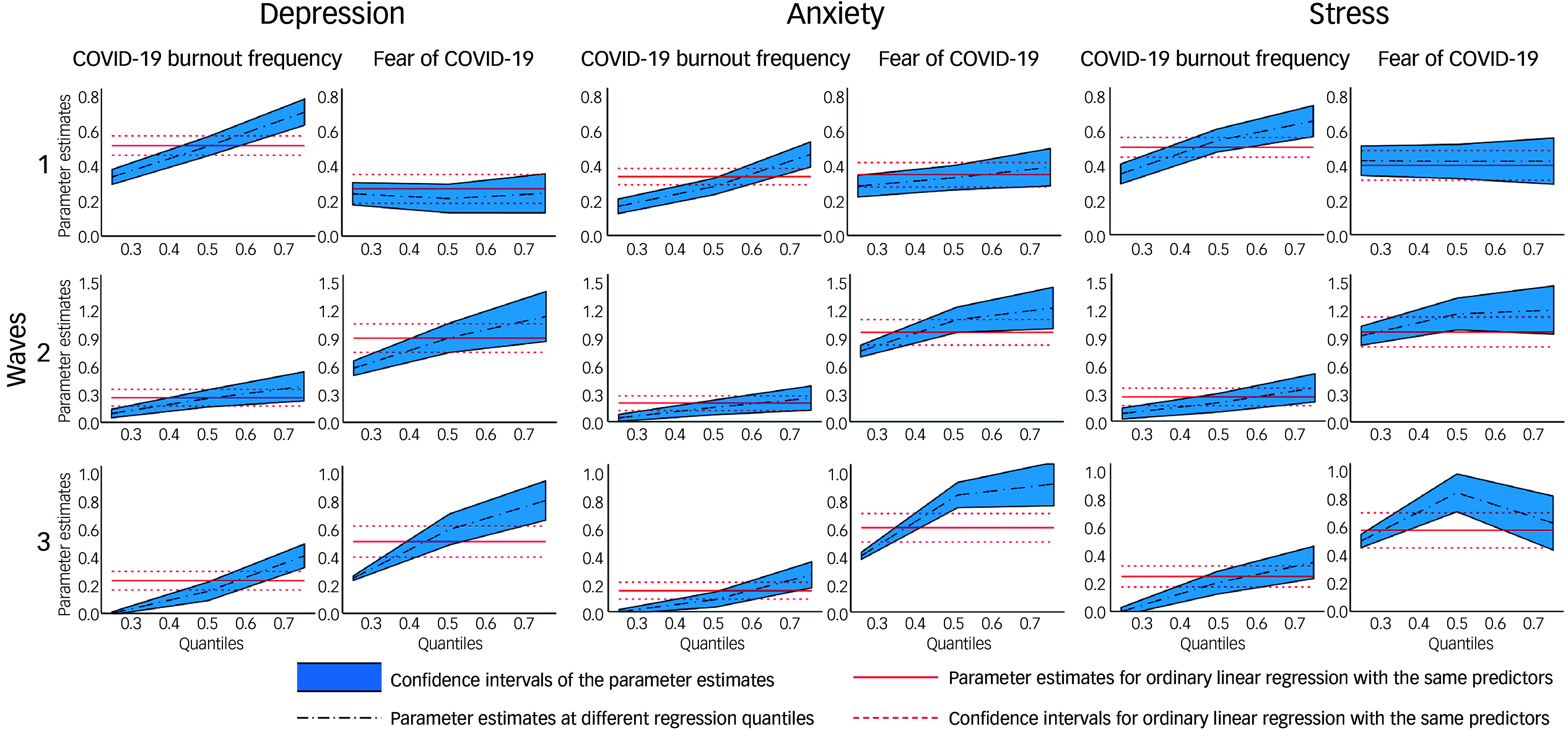
Quantile process plots of depression, anxiety and stress scores across three waves depicting different strengths of association in the 25%, 50% and 75% outcome quantiles.

Between different quantiles of emotional well-being, respondents’ depression, anxiety and stress symptoms in general were incrementally more sensitive at higher quantiles to a change in either pandemic fatigue or pandemic fear, or both, at significant levels. Large regression coefficient differences between waves signified changing strengths of relationship between emotional well-being outcomes, especially in pandemic fatigue and pandemic fear levels. As evidenced by Fig. 3, negative emotional well-being outcomes in Wave 1 were initially strongly predicted by pandemic fatigue, and a weaker quantile-invariant effect of pandemic fears in depression (CV-19 BFS: *B*_25%_ = 0.33 Δ*R* ^2^ = 0.077, *B*_50%_ = 0.52 Δ*R* ^2^ = 0.119, *B*_75%_ = 0.70 Δ*R* ^2^ = 0.154; FCV-19S: *B*_25%_ = 0.25 Δ*R* ^2^ = 0.017, *B*_50%_ = 0.24 Δ*R* ^2^ = 0.013, *B*_75%_ = 0.25 Δ*R* ^2^ = 0.012), anxiety (CV-19 BFS: *B*_25%_ = 0.17 Δ*R* ^2^ = 0.031, *B*_50%_ = 0.28 Δ*R* ^2^ = 0.065, *B*_75%_ = 0.45 Δ*R* ^2^ = 0.094; FCV-19S: *B*_25%_ = 0.28 Δ*R* ^2^ = 0.033, *B*_50%_ = 0.33 Δ*R* ^2^ = 0.041, *B*_75%_ = 0.39 Δ*R* ^2^ = 0.033) and stress (CV-19 BFS: *B*_25%_ = 0.35 Δ*R* ^2^ = 0.075, *B*_50%_ = 0.54 Δ*R* ^2^ = 0.107, *B*_75%_ = 0.63 Δ*R* ^2^ = 0.128; FCV-19S: *B*_25%_ = 0.42 Δ*R* ^2^ = 0.40, *B*_50%_ = 0.42 Δ*R* ^2^ = 0.036, *B*_75%_ = 0.44 Δ*R* ^2^ = 0.023). During Wave 2, the effects of pandemic fears were much more prominent, including for higher quantiles, while pandemic fatigue contributed a much weaker effect on depression (CV-19 BFS: *B*_25%_ = 0.08 Δ*R* ^2^ = 0.008, *B*_50%_ = 0.26 Δ*R* ^2^ = 0.028, *B*_75%_ = 0.38 Δ*R* ^2^ = 0.036; FCV-19S: *B*_25%_ = 0.59 Δ*R* ^2^ = 0.093, *B*_50%_ = 0.90 Δ*R* ^2^ = 0.113, *B*_75%_ = 1.12 Δ*R* ^2^ = 0.119), anxiety (CV-19 BFS: *B*_25%_ = 0.04 Δ*R* ^2^ = 0.004, *B*_50%_ = 0.16 Δ*R* ^2^ = 0.015, *B*_75%_ = 0.25 Δ*R* ^2^ = 0.018; FCV-19S: *B*_25%_ = 0.76 Δ*R* ^2^ = 0.135, *B*_50%_ = 1.09 Δ*R* ^2^ = 0.178, *B*_75%_ = 1.25 Δ*R* ^2^ = 0.182) and stress symptoms (CV-19 BFS: *B*_25%_ = 0.08 Δ*R* ^2^ = 0.007, *B*_50%_ = 0.21 Δ*R* ^2^ = 0.019, *B*_75%_ = 0.38 Δ*R* ^2^ = 0.040; FCV-19S: *B*_25%_ = 0.92 Δ*R* ^2^ = 0.128, *B*_50%_ = 1.13 Δ*R* ^2^ = 0.155, *B*_75%_ = 1.18 Δ*R* ^2^ = 0.127). Wave 3 models illustrated a similar relationship of pandemic fatigue and pandemic fear with emotional well-being, but had significantly declined in goodness-of-fit for the 25% quantile (pseudo *R* ^2^ = 0.028–0.065) and 50% quantile (pseudo *R* ^2^ = 0.141–0.154), including a non-significant effect of pandemic fatigue on the 25% quantile of emotional well-being. Hence, our regression model only retained its goodness-of-fit for the upper tail of predicted DASS-21 scores in Wave 3. Gender, COVID-19 status and beliefs had isolated and inconsistent effects in our models; however, younger ages consistently predict greater risks to emotional well-being at each wave on multiple quantiles.

## Discussion

To our knowledge, this is the first study that attempts to discern the influence of the Chinese dynamic zero-COVID policy on mental health at multiple key policy shifts. Our findings provide evidence that emotional well-being was dynamically influenced by the combination of pandemic fatigue and pandemic fear during the three waves of pivotal policy shifts from the dynamic zero-COVID policy to living-with-COVID policy. Depression, anxiety and stress symptoms were more affected by pandemic fatigue during the peak outbreak of the Omicron variant, whereas the latter two waves during policy shifts towards living with COVID were much more sensitive to pandemic fears.

The first major outbreak of COVID-19 in Hong Kong, measured by Wave 1, took place 2 years into the dynamic zero-COVID strategy and was nevertheless highly evocative of mental health symptoms. This wave markedly induced the highest levels of pandemic fear and pandemic fatigue, and generated the highest level of negative emotional well-being of the waves examined in our study. Subsequently, depression, anxiety and stress symptoms declined until the end of the pandemic, whilst pandemic fatigue and pandemic fear would sharply decline within 10 months at Wave 2, and remain prevalent at Wave 3. Policy Stringency Index data provides us with a standardised, global measure to chart intermediary policy changes between waves, which supplements our findings of changes in pandemic fatigue and pandemic fear levels. Pandemic fatigue is strongly reflected by prolonged adherence to policy stringency levels.^40^ Up until the Omicron outbreak, the Hong Kong population endured PHSMs operating at a consistently high policy stringency under a low incidence of infections. At Wave 1, active cases and policy stringency also peaked in tandem with our other measures, where pandemic fatigue exerted greater strain on emotional well-being than pandemic fear during this wave. In under 2 months, PHSMs were already administered at much less strenuous levels of policy stringency throughout the second Omicron outbreak, before Wave 2. Moderate levels of pandemic fatigue still persisted subsequent to this wave, which we posit may be the effect of policy uncertainty as an alternative source of frustrations with adherence to stringent measures,^41^ given the lack of a clear exit strategy communicated from the government.

Not only were pandemic fatigue levels unchanged between Wave 2 and Wave 3, but so were pandemic fear levels in response to escalating and de-escalating COVID-19 cases of these waves. We adopted the FCV-19S in this study to measure pandemic fear, defined as the perceived risk of contracting COVID-19 by its transmissibility, morbidity and mortality rates.^27^ However, mounting evidence elucidates the complexity of risk perception through a multitude of different factors, including individual pandemic experiences, vaccination rates and beliefs and accuracy of pandemic-related knowledge, as well as agreement with public health strategies.^19,42–44^ The impact of such factors may explain detachments between high fear of COVID-19 levels and risk-prone periods of outbreaks found in other studies.^45,46^

Cui et al^47^ conducted a word frequency analysis during China’s parallel exit from the zero-COVID policy, from a transitional 4-week period before zero-COVID measures were undone, and after, which uncovered that losses of social well-being at the transition period exceeded those in the period of unabated transmissions. Furthermore, compared with Chinese respondents in living-with-COVID countries, lower anxiety and greater resilience towards media misinformation of exaggerated Omicron symptoms were reported abroad, including between Shanghai residents who had just experienced an Omicron outbreak in early 2022, suggesting that coexisting with COVID-19 normalises infection experiences and thus mitigates pandemic fears.^48^ Under Wave 2, the concurrence of extreme fears of COVID-19 and severe negative emotional well-being of respondents (Fig. 2) may denote irrationally negative reactions towards PHSM relaxations in anticipation of an identical resurgence of the Omicron variant in China, or from perceiving an elevated risk of living-with-COVID policies under the Omicron strain. Hence, pandemic fear may have had a pervasive effect on individuals through its interaction with policy uncertainties or disagreement when kept under an elimination policy, affecting emotional well-being more intensively when disengaging PHSMs.^19,47,49^

Winding down from the initial relaxation wave, declining cases and low policy stringency characterised the gap between this and the full relaxation wave, which may have contributed to the loss in effect of extreme fear of COVID-19 on emotional well-being. Our quantile regression model fit had diminished from Waves 1 and 2 for the 25% and 50% quantiles, which could be because of milder reported depression, anxiety and stress symptoms becoming dissociated from a ubiquitous direct relationship with policy-related pandemic fatigue and pandemic fear, and rather the post-pandemic aftereffects of socioeconomic problems, such as financial worries or long-term psychological effects of social distancing, serving as stronger indicators under the post-pandemic setting.^5,50^ For instance, younger respondents have been disproportionately at risk of some negative emotional well-being outcomes during all three waves. Regardless of waning anti-epidemic measures, young adults under academic settings may have experienced a myriad of challenges handling post-pandemic uncertainty in their futures, compounded with socioeconomic worries and negative mental health symptoms over disruptions to their future career plans.^51–53^ Emotional well-being may be exacerbated by apprehensive pandemic fears of this final policy shift as well, given that a lower intolerance to uncertainty has been linked with younger ages.^54^ Implicitly, a slew of unintended effects remains from departing from the zero-COVID strategy. Continued mask wearing in public spaces underscores the cautious attitude towards the policy shift dissolving the mask mandate,^55^ in which many Hong Kong people ascribed mask habits as personal choice and a community-led initiative to combat the pandemic.^10,56^ Pandemic fear has been associated with long-term post-pandemic repercussions for emotional well-being, primarily manifested through an intolerance of uncertainty of future events, which low confidence in government policies reinforces, and may manifest as psychological symptoms of worry tendencies and diminished sleep quality, even in the absence of new outbreaks.^57^

### Strengths and limitations

Our research provides a novel analysis of pandemic fatigue, bridging and incorporating the role of anti-epidemic policy measures as per the extended definition outlined by the World Health Organization (WHO),^6^ which to our knowledge had rarely been integrated as the main focus of current pandemic literature. We achieved this by operationalising ‘pandemic fatigue’ as policy-specific burnout, opting to measure pandemic fatigue using the CV-19 BFS.^25^ We also elucidated the cost of the dynamic zero-COVID policy on emotional well-being, highlighting the predictive power of pandemic fatigue compared to pandemic fear, and demonstrated several uses of interpreting the Policy Stringency Index, especially in the absence of measurements for policy-specific pandemic fatigue. Other strengths include the repeated investigations over three waves capturing the key policy shifts from pandemic peak to living with COVID in the zero-COVID policy context, and the use of validated scales. However, to collect data in response to the unforeseen policy shifts under short time frames, we adopted a convenience and snowball sampling questionnaire design approach primarily through social media, which has given rise to several limitations, such as an unrepresentative sample of younger, active social media users during outbreaks and self-selection bias, limiting the generalisation of the findings. For example, whilst our findings suggest younger age was a risk factor, this direction of effect cannot be generalised to the elderly population (≥65) who were most at risk of COVID-19 fatality but excluded from the scope of our data analysis. Baseline levels of emotional well-being before the effects of new policy measures were unaccounted for, and our sample had not been screened for prior histories of mental health disorders, which may have inflated correlations in higher quantiles of depression, anxiety and stress, or extreme pandemic fears. Moreover, our Wave 2 survey completion rate of 45.7% lagged behind those of Waves 1 and 3, giving rise to potential non-response bias. Collecting self-report data on divisive topics, that is, sentiments towards government policies and vaccination, also poses a risk of social desirability bias. Furthermore, we cannot infer whether policy shift effects on emotional well-being had any long-term effects. Future studies will benefit from a longitudinal design with a representative sample to estimate causal effects of significant events in pandemic, such as policy shifts or new outbreaks.

Policymakers must recognise pandemic fatigue as a hurdle that should be addressed before – not resolved after – policy relaxations. Exemplary public health success with the zero-COVID strategy had the side-effect of rampant vaccine hesitancy, which may become highly resilient under pandemic fatigue.^15^ Thus, policy interventions should tackle pandemic fatigue quickly, such as an early implementation of the Vaccine Pass Initiative that could offer less stringent measures to vaccinated individuals to alleviate the depression, anxiety and stress symptoms of the working-age population and motivate greater vaccine acceptance. Enhancing public communication of the intentions of policy shifts, such as providing a roadmap, could help safeguard emotional well-being from the pandemic fears, bolstered by policy uncertainty and potential COVID-19 misinformation. Greater policy transparency can alleviate psychological impacts of the pandemic without jeopardising the protective factor of anti-epidemic measures. Meanwhile, the outcome-oriented justification of policy shift decisions were insufficient in rectifying the short-term negative emotional well-being effects as found in our study. Overall, greater attentiveness towards assessing psychological costs of pandemic policies could be accomplished by monitoring pandemic fatigue from policy adherence, pandemic fear and policy stringency, which may have further protracted use as predictors of the prevalence of long-term, post-pandemic symptoms.^57^

## Supporting information

Lau et al. supplementary material Lau et al. supplementary material

## Data Availability

The data that support the findings of this study are available from the corresponding author on reasonable request.

## Supplementary material

The supplementary material is available online at https://doi.org/10.1192/bjo.2025.18

## References

[1] Cheng VC-C , Wong S-C , Chuang VW-M , So SY-C , Chen JH-K , Sridhar S , et al. The role of community-wide wearing of face mask for control of coronavirus disease 2019 (COVID-19) epidemic due to SARS-CoV-2. J Infect 2020; 81: 107–14. 32335167 10.1016/j.jinf.2020.04.024 PMC7177146

[2] Gu HG , Xie RP , Adam DC , Tsui JLH , Chu DN , Chang D , et al. Genomic epidemiology of SARS-CoV-2 under an elimination strategy in Hong Kong. Nat Commun 2022; 13: 736. 35136039 10.1038/s41467-022-28420-7 PMC8825829

[3] Choi EPH , Hui BPH , Wan EYF. Depression and anxiety in Hong Kong during COVID-19. Int J Environm Res Publ Health 2020; 17: 3740. 10.3390/ijerph17103740 PMC7277420 32466251

[4] Zhao SZ , Wong JYH , Luk TT , Wai AKC , Lam TH , Wang MP. Mental health crisis under COVID-19 pandemic in Hong Kong, China. Int J Infect Dis 2020; 100: 431–3. 32947051 10.1016/j.ijid.2020.09.030 PMC7492140

[5] Chung GKK , Strong C , Chan YH , Chung RYN , Chen JS , Lin YH , et al. Psychological distress and protective behaviors during the COVID-19 pandemic among different populations: Hong Kong general population, Taiwan healthcare workers, and Taiwan outpatients. Front Med 2022; 9: 800962. 10.3389/fmed.2022.800962 PMC8885588 35242778

[6] Kwok KO , Li KK , Chan HHH , Yi YY , Tang A , Wei WI , Wong SYS. Community responses during early phase of COVID-19 epidemic, Hong Kong. Emerg Infect Dis 2020; 26: 1575–9. 32298227 10.3201/eid2607.200500 PMC7323558

[7] World Health Organization (WHO) Regional Office for Europe. Pandemic Fatigue: Reinvigorating the Public to Prevent COVID-19: Policy Framework for Supporting Pandemic Prevention and Management: Revised Version November 2020. WHO Regional Office for Europe, 2020 (https://www.who.int/europe/publications/i/item/WHO-EURO-2020-1573-41324-56242 [cited 11 Sep 2023]).

[8] Du ZW , Wang L , Shan SW , Lam D , Tsang TK , Xiao JY , et al. Pandemic fatigue impedes mitigation of COVID-19 in Hong Kong. Proc Nat Acad Sci USA 2022; 119: e2213313119. 36417445 10.1073/pnas.2213313119 PMC9860288

[9] Lai DWL , Jin JH , Yan E , Lee VWP. Predictors and moderators of COVID-19 pandemic fatigue in Hong Kong. J Infect Publ Health 2023; 16: 645–50. 10.1016/j.jiph.2023.03.003 PMC9990889 36913768

[10] Li J-B , Lau EYH , Chan DKC. Moral obligation to follow anti-COVID-19 measures strengthens the mental health cost of pandemic burnout. J Affect Disord 2023; 328: 341–4. 36813045 10.1016/j.jad.2023.02.050 PMC9940470

[11] Cheung PHH , Chan CP , Jin DY. Lessons learned from the fifth wave of COVID-19 in Hong Kong in early 2022. Emerg Microb Infect 2022; 11: 1072–8. 10.1080/22221751.2022.2060137 PMC9004509 35348429

[12] Xie R , Edwards KM , Adam DC , Leung KSM , Tsang TK , Gurung S , et al. Resurgence of Omicron BA.2 in SARS-CoV-2 infection-naive Hong Kong. Nat Commun 2023; 14(1): 2422. 37105966 10.1038/s41467-023-38201-5 PMC10134727

[13] Master F. Hong Kong Government Urges Residents Spooked by Citywide Lockdown Not to Panic. Reuters, 2022 (https://www.reuters.com/world/asia-pacific/hong-kong-government-urges-residents-spooked-by-citywide-lockdown-not-panic-2022-03-02/ [cited 11 Sep 2023]).

[14] Luk TT , Zhao SZ , Wu YD , Wong JYH , Wang MP , Lam TH. Prevalence and determinants of SARS-CoV-2 vaccine hesitancy in Hong Kong: a population-based survey. Vaccine 2021; 39: 3602–7. 34034950 10.1016/j.vaccine.2021.05.036 PMC8130539

[15] Bodas M , Kaim A , Velan B , Ziv A , Jaffe E , Adini B. Overcoming the effect of pandemic fatigue on vaccine hesitancy – will belief in science triumph? J Nurs Scholarship 2023; 55: 262–71. 10.1111/jnu.12778 PMC9115056 35388958

[16] Smith DJ , Hakim AJ , Leung GM , Xu WB , Schluter WW , Novak RT , et al. COVID-19 mortality and vaccine coverage – Hong Kong special administrative region, China, January 6, 2022 – March 21, 2022. MMWR Morb Mortal Wkly Rep 2022; 71: 545–8. 35421076 10.15585/mmwr.mm7115e1 PMC9020860

[17] Ioannidis JPA , Zonta F , Levitt M. Estimates of COVID-19 deaths in Mainland China after abandoning zero COVID policy. Eur J Clin Invest 2023; 53: e13956. 36691703 10.1111/eci.13956 PMC10407651

[18] Holbig H. Navigating the dual dilemma between lives, rights and livelihoods: COVID-19 responses in China, Singapore, and South Korea. Z Vgl Politikwissens 2022; 16: 707–31. 10.1007/s12286-023-00555-x PMC9900531 40477846

[19] Lee H , Nam S , Nam EW. Impact of COVID-19 fear on COVID-19 policy support among University students in South Korea. Health Serv Res Manager Epidemiol 2023; 10: 23333928231175801. 10.1177/23333928231175801 PMC10233565 37274356

[20] Abdul RMR , Syed MSN , Tajjudin AI , Roslan N , Jaffar A , Mohideen FB , et al. COVID-19 pandemic fatigue and its sociodemographic, mental health status, and perceived causes: a cross-sectional study nearing the transition to an endemic phase in Malaysia. Int J Environ Res Public Health 2023; 20: 4476. 36901486 10.3390/ijerph20054476 PMC10001764

[21] Centre of Health Protection. Latest Situation of Reported Cases COVID-19. Centre of Health Protection, 2023 (https://www.chp.gov.hk/files/misc/latest_situation_of_reported_cases_covid_19_eng.csv [cited 12 Sep 2023]).

[22] Worldometer. Coronavirus Case Statistics in China, Hong Kong SAR. Worldometer, 2023 (https://www.worldometers.info/coronavirus/country/china-hong-kong-sar/ [cited 12 Sep 2023]).

[23] Hale T , Angrist N , Goldszmidt R , Kira B , Petherick A , Phillips T , et al. A global panel database of pandemic policies (Oxford COVID-19 Government Response Tracker). Nat Hum Behav 2021; 5: 529–38. 33686204 10.1038/s41562-021-01079-8

[24] STROBE Statement. Strengthening the Reporting of Observational Studies in Epidemiology. STROBE Statement, 2023 (http://www.strobe-statement.org/ [cited 11 Nov 2023]).

[25] Lau SSS , Ho CCY , Pang RCK , Su SS , Kwok H , Fung SF , Ho RC. COVID-19 burnout subject to the dynamic zero-COVID policy in Hong Kong: development and psychometric evaluation of the COVID-19 burnout frequency scale. Sustainability 2022; 14: 8235.

[26] Cureton EE. The upper and lower twenty-seven per cent rule. Psychometrika 1957; 22: 293–6.

[27] Ahorsu DK , Lin CY , Imani V , Saffari M , Griffiths MD , Pakpour AH. The fear of COVID-19 scale: development and initial validation. Int J Ment Health Addict 2022; 20: 1537–45. 32226353 10.1007/s11469-020-00270-8 PMC7100496

[28] Chi XL , Chen SY , Chen YY , Chen DY , Yu Q , Guo TY , et al. Psychometric evaluation of the fear of COVID-19 scale among Chinese population. Int J Ment Health Addict 2022; 20: 1273–88. 33456407 10.1007/s11469-020-00441-7 PMC7799163

[29] Ochnik D , Rogowska AM , Benatov J , Arzensek A. Adaptation and preliminary validation of the fear of coronavirus vaccination scale in the prospective study among a representative sample of Polish, Israeli, Slovenian, and German Adults during the COVID-19 pandemic. Int J Environ Res Public Health 2022; 19: 11587. 36141859 10.3390/ijerph191811587 PMC9517357

[30] Yang WQ , Li P , Huang YB , Yang X , Mu W , Jing WW , et al. Cross-cultural adaptation and validation of the fear of COVID-19 scale for Chinese university students: a cross-sectional study. Int J Environ Res Public Health 2022; 19: 8624. 35886480 10.3390/ijerph19148624 PMC9320396

[31] Antony MM , Bieling PJ , Cox BJ , Enns MW , Swinson RP. Psychometric properties of the 42-item and 21-item versions of the Depression Anxiety Stress Scales in clinical groups and a community sample. Psycholog Assess 1998; 10: 176–81.

[32] Lovibond SH , Lovibond PF . Manual for the Depression Anxiety Stress Scales 2nd ed. Psychology Foundation of Australia, 1995.

[33] Brown TA , Chorpita BF , Korotitsch W , Barlow DH. Psychometric properties of the Depression Anxiety Stress Scales (DASS) in clinical samples. Behav Res Therapy 1997; 35: 79–89. 10.1016/s0005-7967(96)00068-x 9009048

[34] Henry JD , Crawford JR. The short-form version of the Depression Anxiety Stress Scales (DASS-21): construct validity and normative data in a large non-clinical sample. Br J Clin Psychol 2005; 44: 227–39. 16004657 10.1348/014466505X29657

[35] Moussa MT , Lovibond PF , Laube R. Psychometric Properties of a Chinese Version of the 21-Item Depression Anxiety Stress Scales (DASS21). Transcultural Mental Health Centre, Cumberland Hospital, 2001.

[36] Wilcox RR. ANCOVA. In Introduction to Robust Estimation and Hypothesis Testing (Fifth Edition) (ed R Wilcox ): 773–80. Academic Press, 2021.

[37] Morales-Oñate V , Morales-Oñate B. MTest: a bootstrap test for multicollinearity. Rev Politéc 2023; 51: 53–62.

[38] Wilcox RR , Tian TS. Measuring effect size: a robust heteroscedastic approach for two or more groups. J Appl Stat 2011; 38: 1359–68.

[39] Koenker R , Machado JAF. Goodness of fit and related inference processes for quantile regression. J Am Stat Assoc 1999; 94(448): 1296–310.

[40] Jorgensen F , Bor A , Rasmussen MS , Lindholt MF , Petersen MB. Pandemic fatigue fueled political discontent during the COVID-19 pandemic. Proc Nat Acad Sci USA 2022; 119: e2201266119. 36413499 10.1073/pnas.2201266119 PMC9860270

[41] Haktanir A , Can N , Seki T , Kurnaz MF , Dilmac B. Do we experience pandemic fatigue? Current state, predictors, and prevention. Curr Psychol 2022; 41: 7314–25. 34690475 10.1007/s12144-021-02397-w PMC8527300

[42] Robinson E , Daly M. Explaining the rise and fall of psychological distress during the COVID-19 crisis in the United States: longitudinal evidence from the understanding America study. Br J Health Psychol 2021; 26: 570–87. 33278066 10.1111/bjhp.12493

[43] Hussong J , Möhler E , Kühn A , Wenning M , Gehrke T , Burckhart H , et al. Mental health and health-related quality of life in German adolescents after the third wave of the COVID-19 pandemic. Children-Basel 2022; 9: 780. 35740717 10.3390/children9060780 PMC9221692

[44] Duong HT , Sun YX , Nguyen LTV , Nguyen KT , Popova L. Before Omicron’s arrival: effects of negative emotions and comparative optimism on COVID-19 protection and detection behaviors. Health Commun 2023; 39: 1429–43. 37264526 10.1080/10410236.2023.2218141

[45] Peng XD , Liu LL , Liang SW , Chen JB , Zhao JB. Longitudinal changes in fear and anxiety among Chinese college students during the COVID-19 pandemic: a one-year follow-up study. Curr Psychol 2024; 43: 13887-96. 10.1007/s12144-022-03487-z PMC9323878 35910238

[46] Nagib N , Horita R , Miwa T , Adachi M , Tajirika S , Imamura N , et al. Impact of COVID-19 on the mental health of Japanese university students (years II–IV). Psychiatry Res 2023; 325: 115244. 37182282 10.1016/j.psychres.2023.115244

[47] Cui ZH , Liu L , Zhuang CC. The impact of the removal of zero-COVID policies on subjective well-being: evidence from a digital world. Econ Lett 2023; 229: 111189.

[48] Shan D , Liu C , Li SY , Zheng YD. Increased anxiety from fear of Omicron in China as compared to North America and Western Europe: a cross-sectional Kendall’s tau-b analysis using the generalized anxiety disorder 7-item questionnaire. Front Psychiatry 2022; 13: 977361. 36111310 10.3389/fpsyt.2022.977361 PMC9468740

[49] Aknin L , Andretti B , Goldszmidt R , Helliwell J , Petherick A , Neve J-E , et al. Policy stringency and mental health during the COVID-19 pandemic: a longitudinal analysis of data from 15 countries. Lancet Publ Health 2022; 7: e417–26. 10.1016/S2468-2667(22)00060-3 PMC9023007 35461592

[50] Jamshaid S , Bahadar N , Jamshed K , Rashid M , Afzal MI , Tian L , et al. Pre- and post-pandemic (COVID-19) mental health of international students: data from a longitudinal study. Psychol Res Behav Manag 2023; 16: 431–46. 36814636 10.2147/PRBM.S395035 PMC9939801

[51] Jung J , Horta H , Postiglione GA. Living in uncertainty: the COVID-19 pandemic and higher education in Hong Kong. Stud High Educ 2021; 46: 107–20.

[52] Kim HJ , Meeker TJ , Tulloch IK , Mullins J , Park J-H , Bae SH. Pandemic fatigue and anxiety sensitivity as associated factors with posttraumatic stress symptoms among university students in South Korea during the prolonged COVID-19 pandemic. Int J Publ Health 2022; 67: 1604552. 10.3389/ijph.2022.1604552 PMC9137407 35645697

[53] Zhou TS , Bao YC , Guo DF , Bai YP , Wang RZ , Cao XY , et al. Intolerance of uncertainty and future career anxiety among Chinese undergraduate students during COVID-19 period: fear of COVID-19 and depression as mediators. Front Public Health 2022; 10: 1015446. 36523580 10.3389/fpubh.2022.1015446 PMC9745131

[54] Basevitz P , Pushkar D , Chaikelson J , Conway M , Dalton C. Age-related differences in worry and related processes. Int J Aging Hum Dev 2008; 66: 283–305. 18507331 10.2190/AG.66.4.b

[55] Jett J , Gupta K , Le S. On Its First Day Free of Masks, Hong Kong is in No Rush to Take Them Off. NBC News, 2023 (https://www.nbcnews.com/news/world/hong-kong-lifts-covid-mask-mandate-rcna72641 [cited 11 Sep 2023]).

[56] Cheung T , Wong N. Coronavirus: Hong Kong Residents Unhappy with Covid-19 Response – and Surgical Masks One Big Reason Why, Post Survey Shows. SCMP, 2020 (https://www.scmp.com/news/hong-kong/politics/article/3077761/coronavirus-post-poll-shows-hong-kong-residents-unhappy [cited 7 Sep 2023]).

[57] Quigley M , Whiteford S , Cameron G , Zuj DV , Dymond S. Longitudinal assessment of COVID-19 fear and psychological wellbeing in the United Kingdom. Journal of Health Psychology 2023; 28: 726–38. 36397647 10.1177/13591053221134848 PMC9679309

