# Supplementary material for: Psychological cost of Hong Kong’s zero-COVID policy: three-wave repeated cross-sectional study of pandemic fatigue, pandemic fear and emotional well-being from peak pandemic to living-with-COVID policy shift
Source: BJPsych Open. 2025 Mar 24;11(2):e68. doi: 10.1192/bjo.2025.18 (PMC12001963; doi:10.1192/bjo.2025.18)
Supplement: Lau et al. supplementary material [file S2056472425000183sup001.docx]

**Psychological cost of Hong Kong’s zero-COVID policy: A three-wave repeated cross-sectional study of pandemic fatigue, pandemic fear and emotional well-being from peak pandemic to living with COVID policy shift**

Sam SS Lau, Jason WL Fong, Marco CH Cheng

**Supplementary Materials**

[**Appendix 1 1**](#_Toc185496724)

[Supplementary Text 1: Measures of policy stringency](#_Toc185496725)

[**Appendix 2 3**](#_Toc185496726)

[Supplementary Table 1. Descriptive statistics, internal validity and test of normality of scales](#_Toc185496727)

[**Appendix 3 4**](#_Toc185496728)

[Supplementary Figure 1. Boxplots of dependent and independent variable scales](#_Toc185496729)

[Supplementary Figure 2. Normal Q-Q plots and histograms of scale scores](#_Toc185496730)

[**Appendix 4 6**](#_Toc185496731)

[Supplementary Table 2. 20% trimmed mean scores of emotional well-being under different levels of burnout frequency and fear of COVID-19](#_Toc185496732)

[**Appendix 5 7**](#_Toc185496733)

[Supplementary Table 3. Pairwise comparisons of 20% trimmed mean differences between waves when controlling for gender and age](#_Toc185496734)

[**Appendix 6 9**](#_Toc185496735)

[Supplementary Table 4. Correlation matrix of scales](#_Toc185496736)

[Supplementary Table 5. Bootstrapped multicollinearity test of pandemic fatigue, pandemic fears, demographic factors, and COVID-19 status and beliefs as predictors of emotional well-being](#_Toc185496737)

[**Appendix References 11**](#_Toc185496738)

# Appendix 1:

# Supplementary Text 1: Measures of policy stringency


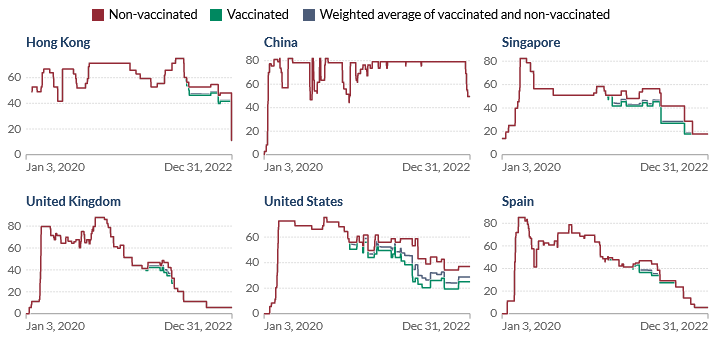

*Stringency Index of Hong Kong and 5 other countries provided by data from the OxCGRT project.* *Retrieved from:* [*https://ourworldindata.org/covid-stringency-index*](https://ourworldindata.org/covid-stringency-index) *on 18 July 2023.*

Aknin et al.^1^ details two distinct styles of anti-epidemic policies enacted by 15 different countries—elimination and mitigation strategies— which correspond to policies that aim to either prevent local transmissions of the virus with targeted measures, i.e. zero-COVID strategies widely used in Asia, or policies that try to control the spread of the virus with overarching measures without only isolating cases, e.g. in the UK, US and Spain. The Oxford Coronavirus Government Response Tracker (OxCGRT) charts the ‘stringency index’ of over 180 individual countries as a composite metric of the intensity of government policies and responses enacted to contain the spread of the COVID-19 pandemic from the start of the pandemic until the end of 2022 through nine indicators: (1) school closures, (2) workplace closures, (3) cancellation of public events, (4) restrictions on public gathering sizes, (5) public transport closures, (6) stay-at-home requirements, (7) restrictions on the local movement of people, (8) restrictions on international travel, and (9) public information campaigns.^2^ Hong Kong is observed to have been able to utilize less stringent measures compared to many other countries. At a peak stringency of 75 during the start of the fifth wave of the pandemic on 15 March 2022, other countries, including those using similar policies, have exceeded Hong Kong since the start of the pandemic by March-to-April 2020. In general, countries using elimination strategies early have required less stringent policies on average due to their success at the early stages of the pandemic. Over the course of the pandemic, Hong Kong did not implement the most stringent countermeasures, such as the liberal use of city-wide lockdowns in countries such as mainland China, Australia and the UK, but Hong Kong’s dynamic zero-COVID strategy still maintained one of longest running, and consistently high stringency policies in the world in a similar fashion to China.


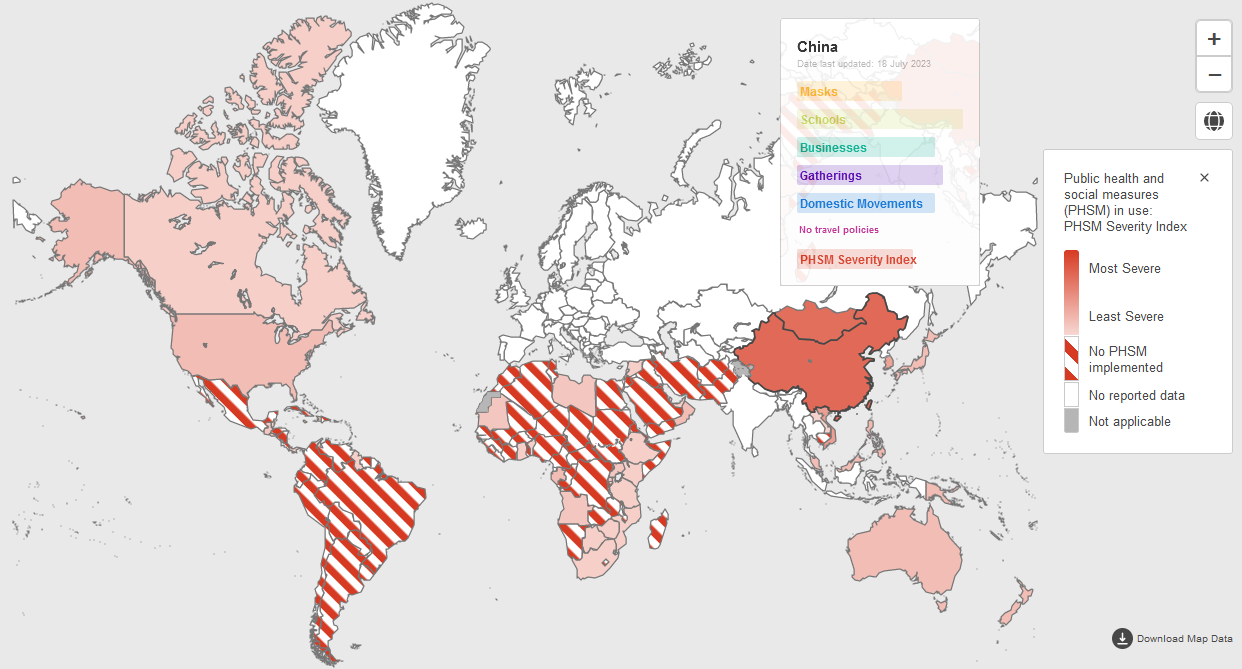

*Note: Respective PHSM Severity Indexes of countries provided by WHO (2023) data. Data on Hong Kong is not recorded, and not included as part of China. Retrieved from:* [*https://covid19.who.int/region/wpro/country/cn/measures*](https://covid19.who.int/region/wpro/country/cn/measures) *on 18 July 2023.*

Another global measure of relative policy stringency was the ‘PHSM Severity Index’, developed by WHO Regional Office of Europe^3^, which was developed after the Stringency Index scale and shares an average of 80.9% in similarity scores. The main distinction that this metric has to the OxCGRT stringency index is the omission of a broad range of indicators, such as government containment and closure policies, economic policies and health system policies, choosing to focus more on 6 key PHSMs: (1) mask wearing, (2) school closures, (3) business closures, (4) gathering restrictions, (5) domestic movement restrictions and (6) international travel restrictions. As such, this metric places China and Mongolia as the only countries under the “most severe” degrees of PHSMs implemented. Although Hong Kong was excluded from having its own PHSM Severity Index, we still can make inferences based on how similar both dynamic zero-COVID strategies were in their core anti-epidemic tactics, barring China’s capacity to implement city-wide lockdowns. Based on this, we can posit that Hong Kong’s zero-COVID policy measures have a strong chance of directly and universally affecting the mental and emotional well-being of Hong Kong residents, given the severity and the long-term exposure to these PHSMs and a greater chance of disrupting the normalcy of many Hong Kong residents’ daily lives.

# Appendix 2:

# Supplementary Table 1. Descriptive statistics, internal validity and test of normality of scales

| Wave | Scales | *M* | *SD* | Skew | Kurtosis | *a* | ω | K-S statistic |
| --- | --- | --- | --- | --- | --- | --- | --- | --- |
| Peak Outbreak Wave (Wave 1) | CV–19 BFS | 20.03 | 8.91 | 0.04 | -1.14 | 0.90 | 0.91 | 0.074*** |
|  | FCV–19S | 16.41 | 5.54 | 0.41 | 0.04 | 0.89 | 0.88 | 0.075*** |
|  | DASS-21 - Depression | 10.11 | 9.15 | 1.10 | 0.95 | 0.88 | 0.89 | 0.135*** |
|  | DASS-21 - Anxiety | 7.79 | 7.44 | 1.28 | 1.75 | 0.82 | 0.82 | 0.160*** |
|  | DASS-21 - Stress | 12.09 | 9.29 | 0.75 | 0.12 | 0.88 | 0.89 | 0.104*** |
| Initial Relaxation  Wave (Wave 2) | CV-19 BFS | 15.24 | 7.59 | 0.84 | -0.11 | 0.89 | 0.89 | 0.138*** |
|  | FCV-19S | 10.71 | 4.34 | 1.69 | 4.12 | 0.90 | 0.90 | 0.197*** |
|  | DASS-21 - Depression | 7.52 | 8.71 | 1.34 | 1.41 | 0.91 | 0.91 | 0.194*** |
|  | DASS-21 - Anxiety | 7.08 | 7.71 | 1.43 | 2.08 | 0.85 | 0.85 | 0.179*** |
|  | DASS-21 - Stress | 8.61 | 8.87 | 1.25 | 1.40 | 0.91 | 0.91 | 0.166*** |
| Mask Mandate Lift Wave (Wave 3) | CV-19 BFS | 15.27 | 8.25 | 0.78 | -0.23 | 0.90 | 0.90 | 0.124*** |
|  | FCV-19S | 10.77 | 4.82 | 1.89 | 4.83 | 0.92 | 0.92 | 0.217*** |
|  | DASS-21 - Depression | 6.05 | 7.61 | 1.52 | 2.03 | 0.88 | 0.89 | 0.213*** |
|  | DASS-21 - Anxiety | 5.96 | 7.00 | 1.49 | 2.03 | 0.85 | 0.85 | 0.197*** |
|  | DASS-21 - Stress | 7.83 | 8.43 | 1.25 | 1.26 | 0.89 | 0.89 | 0.176*** |

*M* = *Mean;* *SD* = *Standard deviation;* *a = Cronbach’s alpha; ω = McDonald’s omega; K-S statistic = Kolmogorov-Smirnov statistic; CV-19 BFS = COVID-19 Burnout Frequency Scale; FCV-19S = Fear of COVID-19 Scale; DASS-21 = Depression, Anxiety, Stress Scale with 21 items; *** = p<0.001.
Note:* Cronbach’s alpha demonstrates that all scales had good or excellent internal validity. A Kolmogorov-Smirnov statistic was significant for all scales in all 3 waves, indicating that our scale responses had non-parametric distributions.

# Appendix 3:

# Supplementary Figure 1. Boxplots of dependent and independent variable scales

*
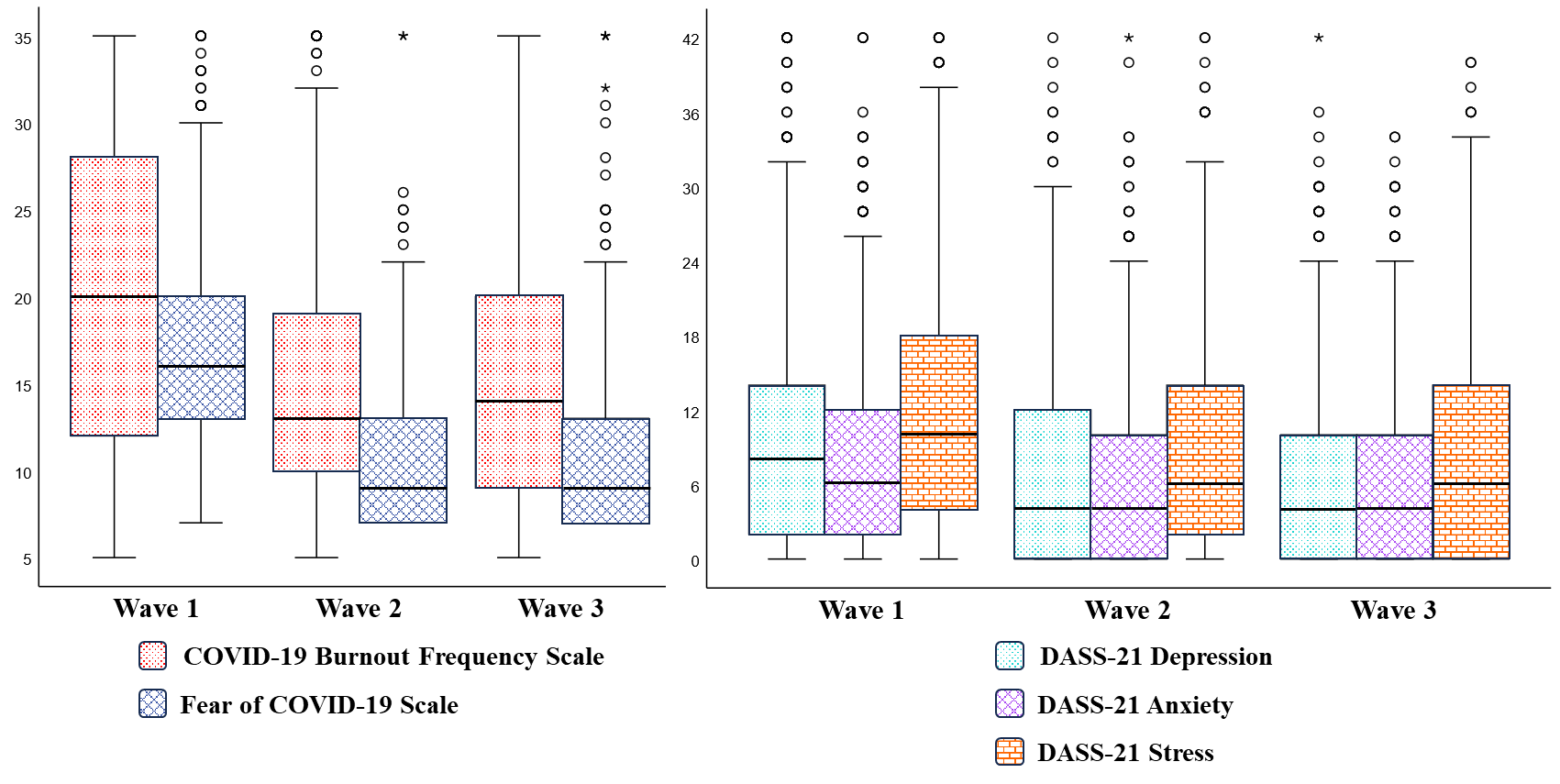
*
*Note:* The ranges of scale scores were 5–35 and 7–35 for COVID-19 Burnout Frequency Scale and Fear of COVID-19 Scale, respectively. The Depression Anxiety Stress Scale-21 subscales ranged from 0–42. Our data featured several positively skewed distributions of scale scores, given the lower median scores than the means in particular for the Fear of COVID-19 Scale, DASS-21 Depression and Anxiety in Waves 2 and 3. Outliers in our data consisted of high scale scores only.

# Supplementary Figure 2. Normal Q-Q plots and histograms of scale scores

*
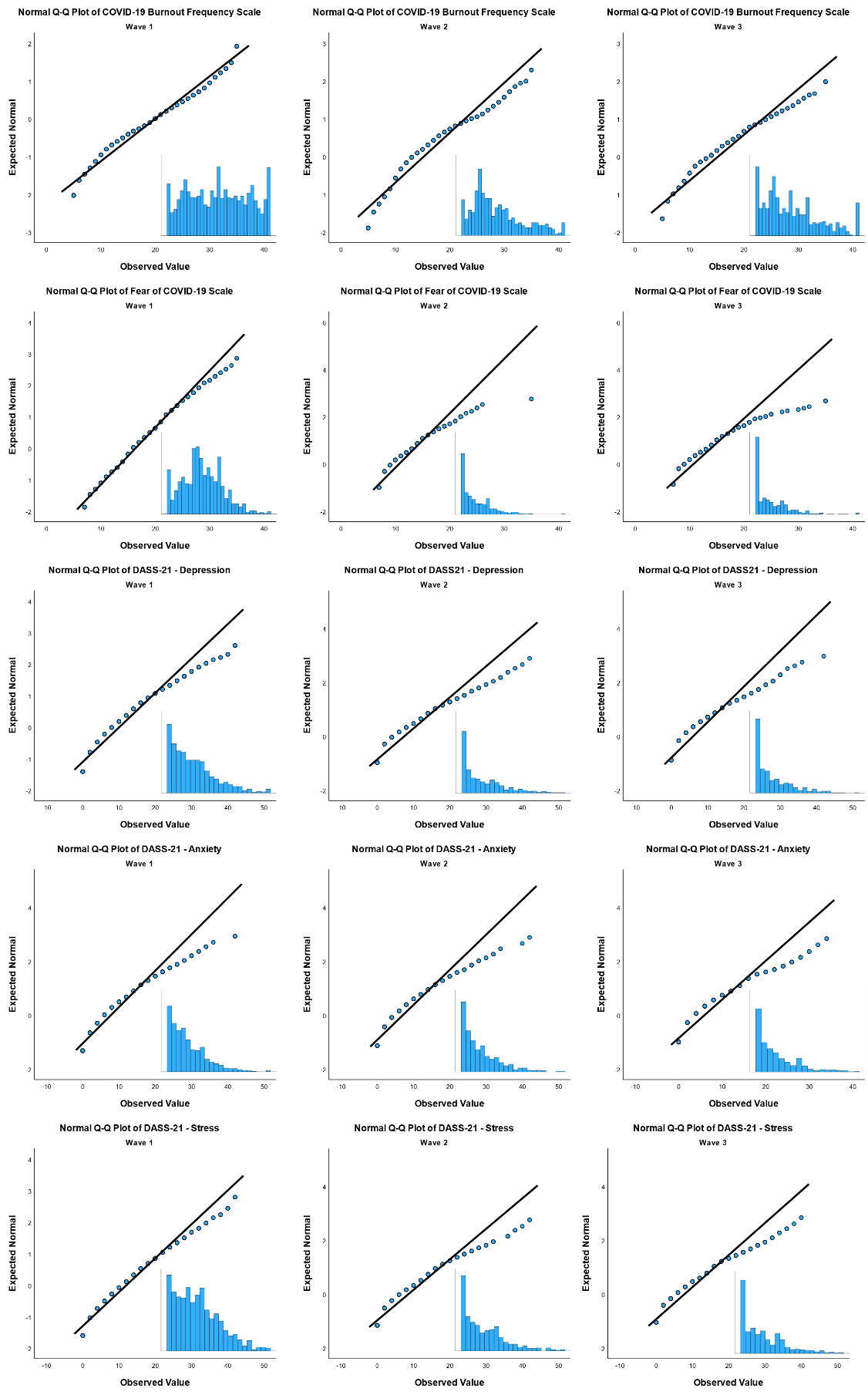

Note*: Deviations in the perfect positive correlation line between the upper end of expected normal values from observed values indicates a positive skew for the Fear of COVID-19 Scale and DASS-21 scores, where floor effects can be seen in the histograms of DASS-21 scores at each wave.

# Appendix 4:

# Supplementary Table 2. 20% trimmed mean scores of emotional well-being under different levels of burnout frequency and fear of COVID-19

|  | Depression | | | Anxiety | | | Stress | | |
| --- | --- | --- | --- | --- | --- | --- | --- | --- | --- |
| Scale | Wave 1 | Wave 2 | Wave 3 | Wave 1 | Wave 2 | Wave 3 | Wave 1 | Wave 2 | Wave 3 |
|  | M_t_ (SD_t_) | | | M_t_ (SD_t_) | | | M_t_ (SD_t_) | | |
| CV-19 BFS |  |  |  |  |  |  |  |  |  |
| Low  (5 - 12) | 2.50 (3.40) | 2.86 (4.63) | 2.06 (3.23) | 2.52 (3.30) | 3.30 (4.36) | 2.82 (4.20) | 4.86 (6.31) | 4.25 (5.89) | 3.85 (5.19) |
| Middle (13 - 25) | 8.26 (7.99) | 6.69 (7.77) | 5.64 (7.34) | 6.38 (6.03) | 6.56 (7.16) | 5.71 (7.69) | 10.89 (9.48) | 8.20 (8.41) | 8.13 (9.33) |
| High (26 - 35) | 15.63 (10.80) | 11.91 (13.58) | 7.17 (9.46) | 10.90 (9.62) | 10.00 (10.58) | 5.66 (7.78) | 17.30 (10.34) | 13.12 (14.54) | 8.68 (10.30) |
| FCV-19s |  |  |  |  |  |  |  |  |  |
| Low (7 - 16) | 7.42 (8.29) | 4.51 (6.43) | 3.30 (4.94) | 5.00 (5.76) | 4.44 (5.22) | 3.54 (4.97) | 9.17 (8.76) | 5.96 (7.67) | 5.32 (7.43) |
| Extreme (17 - 35) | 10.09 (9.40) | 18.08 (12.19) | 11.89 (11.84) | 8.52 (8.17) | 17.83 (9.23) | 12.40 (11.17) | 13.56 (9.56) | 20.25 (10.52) | 13.83 (12.69) |

*Note: M_t_* = 20% Trimmed Mean; *SD_t_* = 20% Trimmed Standard Deviation; CV-19 BFS = COVID-19 Burnout Frequency Scale; FCV-19s = Fear of COVID-19 Scale; DASS-21 = Depression, Anxiety, Stress Scale with 21 items. Extreme FCV-19s scores coincided with moderate-to-severe DASS-21 scores during Wave 2 only.

# Appendix 5:

# Supplementary Table 3. Pairwise comparisons of 20% trimmed mean differences between waves when controlling for gender and age

| Factor | Wave 1 vs. 2 | | | Wave 2 vs. 3 | | | Wave 1 vs. 3 | | |
| --- | --- | --- | --- | --- | --- | --- | --- | --- | --- |
|  | MD_t_ | Adjusted *p* | Effect Size (ξ^2^) | MD_t_ | Adjusted *p* | Effect Size (ξ^2^) | MD_t_ | Adjusted *p* | Effect Size (ξ^2^) |
| Low Burnout x Depression | -0.36 | 0.971 | 0.037 | 0.80 | 0.011–0.660 | 0.055 | 0.44 | 0.004–0.665 | 0.032 |
| Low Burnout x Anxiety | -0.78 | 0.776 | 0.030 | 0.48 | 0.081–0.810 | 0.046 | -0.30 | 0.125–0.467 | 0.021 |
| Low Burnout x Stress | 0.62 | <0.793–0.803 | 0.040 | 0.39 | 0.154–0.733 | 0.052 | 1.01 | 0.061–0.622 | 0.040 |
| Medium Burnout x Depression | 1.56 | 0.008–0.418 | 0.071 | 1.05 | 0.358–0.994 | 0.036 | 2.62 | <0.001–0.271 | 0.033 |
| Medium Burnout x Anxiety | -0.18 | 0.946 | 0.033 | 0.85 | 0.438–0.763 | 0.028 | 0.67 | 0.483–0.606 | 0.025 |
| Medium Burnout x Stress | 2.69 | 0.001–0.292 | 0.093 | 0.076 | 0.604–0.877 | 0.040 | 2.77 | <0.001–0.290 | 0.024 |
| High Burnout x Depression | 3.73 | 0.038–0.956 | 0.117 | 4.74 | 0.008–0.334 | 0.079 | 8.46 | <0.001–0.058 | 0.117 |
| High Burnout x Anxiety | 0.90 | 0.253–0.271 | 0.094 | 4.34 | 0.010–0.669 | 0.083 | 5.24 | 0.008–0.207 | 0.081 |
| High Burnout x Stress | 4.18 | 0.252–0.463 | 0.106 | 4.44 | 0.021–0.605 | 0.065 | **8.62** | <0.002–0.039 | 0.103 |
| Extreme Fear of COVID-19 x Depression | **-8.32** | 0.003–0.013 | 0.110 | 6.39 | 0.078–0.234 | 0.100 | 2.36 | 0.405 | 0.057 |
| Extreme Fear of COVID-19 x Anxiety | **-9.69** | <0.001 | 0.176 | 6.79 | 0.057–0.107 | 0.124 | 4.39 | 0.059–0.198 | 0.069 |
| Extreme Fear of COVID-19 x Stress | **-7.28** | 0.010–0.038 | 0.106 | **8.20** | 0.022–0.027 | 0.123 | 0.46 | 0.900 | 0.056 |
| Low Fear of COVID-19 x Depression | **3.76** | <0.001 | 0.171 | **1.84** | 0.008–0.027 | 0.085 | **-3.77** | <0.001 | 0.141 |
| Low Fear of COVID-19 x Anxiety | 1.15 | 0.020–0.716 | 0.104 | 1.20 | 0.066–0.218 | 0.084 | -1.47 | <0.001–0.507 | 0.085 |
| Low Fear of COVID-19 x Stress | **3.88** | <0.001 | 0.144 | 0.95 | 0.130–0.394 | 0.065 | **-3.67** | <0.001–0.001 | 0.109 |

*Note: MD_t_* = 20% trimmed mean differences. Significant mean differences at all covariate group levels are displayed as **bold**. Effect size was measured by the explanatory measure of effect size (ξ^2^), generalised for use on non-parametric and unequal sample size data. Small effect size: ξ^2^ = 0.14; medium effect size: ξ^2^ = 0.34; large effect size: ξ^2^ = 0.52. Analysis was conducted using the WRS package in R.^4^

# Appendix 6:

# Supplementary Table 4. Correlation matrix of scales

| Wave | Scale | 1 | 2 | 3 | 4 | 5 |
| --- | --- | --- | --- | --- | --- | --- |
| Peak Outbreak Wave (Wave 1) | 1. CV–19 BFS | 1 |  |  |  |  |
|  | 2. FCV–19s | 0.108*** | 1 |  |  |  |
|  | 3. DASS-21 – Depression | 0.566*** | 0.194*** | 1 |  |  |
|  | 4. DASS-21 – Anxiety | 0.449*** | 0.293*** | 0.722*** | 1 |  |
|  | 5. DASS-21 – Stress | 0.518*** | 0.279*** | 0.789*** | 0.789*** | 1 |
| Initial Relaxation  Wave (Wave 2) | 1. CV–19 BFS | 1 |  |  |  |  |
|  | 2. FCV–19s | 0.199*** | 1 |  |  |  |
|  | 3. DASS-21 – Depression | 0.326*** | 0.468*** | 1 |  |  |
|  | 4. DASS-21 – Anxiety | 0.295*** | 0.531*** | 0.803*** | 1 |  |
|  | 5. DASS-21 – Stress | 0.315*** | 0.522*** | 0.837*** | 0.839*** | 1 |
| Mask Mandate Lift Wave (Wave 3) | 1. CV–19 BFS | 1 |  |  |  |  |
|  | 2. FCV–19s | 0.184*** | 1 |  |  |  |
|  | 3. DASS – Depression | 0.319*** | 0.385*** | 1 |  |  |
|  | 4. DASS-21 – Anxiety | 0.247*** | 0.441*** | 0.818*** | 1 |  |
|  | 5. DASS-21 – Stress | 0.306*** | 0.406*** | 0.842*** | 0.831*** | 1 |

*Note:* CV-19 BFS = COVID-19 Burnout Frequency Scale; FCV-19s = Fear of COVID-19 Scale; DASS-21 = Depression, Anxiety, Stress Scale-21 *** *p* < 0.001.
Note: Correlation coefficients were given by Spearman’s rho. CV-19 BFS and FCV-19s showed weak correlations relative to DASS-21 categories, which the latter were all strongly correlated.

# Supplementary Table 5. Bootstrapped multicollinearity test of pandemic fatigue, pandemic fears, demographic factors, and COVID-19 status and beliefs as predictors of emotional well-being

| Variables |  | Depression | | | Anxiety | | | Stress | | |
| --- | --- | --- | --- | --- | --- | --- | --- | --- | --- | --- |
|  | VIF | *R^2^*_auxiliary_ | *R^2^*_global_ | | *R^2^*_auxiliary_ | *R^2^*_global_ | | *R^2^*_auxiliary_ | *R^2^*_global_ | |
| **Wave 1**: |  |  | |  |  | |  |  | |  |
| CV-19 BFS | 1.29*** | 0.233*** | | 0.353 | 0.233*** | | 0.298 | 0.233*** | | 0.334 |
| FCV-19s | 1.12*** | 0.112*** | |  | 0.112*** | |  | 0.112*** | |  |
| Gender | 1.02*** | 0.024*** | |  | 0.024*** | |  | 0.024*** | |  |
| Age | 1.65*** | 0.398 | |  | 0.398 | |  | 0.398 | |  |
| Employed | 3.48*** | 0.715 | |  | 0.715 | |  | 0.715 | |  |
| Student | 4.24*** | 0.766 | |  | 0.766 | |  | 0.766 | |  |
| No. of Vaccinations taken | 1.13*** | 0.121*** | |  | 0.121*** | |  | 0.121*** | |  |
| Contracted COVID-19 before | 1.04*** | 0.044*** | |  | 0.044*** | |  | 0.044*** | |  |
| Herd Immunity outlook | 1.29*** | 0.229** | |  | 0.229** | |  | 0.229** | |  |
| **Wave 2**: |  |  | |  |  | |  |  | |  |
| CV-19 BFS | 1.12*** | 0.120*** | | 0.393 | 0.120*** | | 0.425 | 0.120*** | | 0.375 |
| FCV-19s | 1.07*** | 0.083*** | |  | 0.083*** | |  | 0.083*** | |  |
| Gender | 1.07*** | 0.081*** | |  | 0.081*** | |  | 0.081*** | |  |
| Age | 1.75*** | 0.441 | |  | 0.441 | |  | 0.441 | |  |
| Employed | 6.88 | 0.857 | |  | 0.857 | |  | 0.857 | |  |
| Student | 8.12 | 0.879 | |  | 0.879 | |  | 0.879 | |  |
| No. of Vaccinations taken | 1.05*** | 0.062*** | |  | 0.062*** | |  | 0.062*** | |  |
| Contracted COVID-19 before | 1.05*** | 0.060*** | |  | 0.060*** | |  | 0.060*** | |  |
| Herd Immunity outlook | 1.02*** | 0.039*** | |  | 0.039*** | |  | 0.039*** | |  |
| **Wave 3**: |  |  | |  |  | |  |  | |  |
| CV-19 BFS | 1.17*** | 0.154** | | 0.260 | 0.154** | | 0.279 | 0.154* | | 0.233 |
| FCV-19s | 1.08*** | 0.086*** | |  | 0.086*** | |  | 0.086*** | |  |
| Gender | 1.04*** | 0.055*** | |  | 0.055*** | |  | 0.055*** | |  |
| Age | 1.92*** | 0.485 | |  | 0.485 | |  | 0.485 | |  |
| Employed | 2.67*** | 0.631 | |  | 0.631 | |  | 0.631 | |  |
| Student | 3.79*** | 0.740 | |  | 0.740 | |  | 0.740 | |  |
| No. of Vaccinations taken | 1.06*** | 0.068*** | |  | 0.068*** | |  | 0.068*** | |  |
| Contracted COVID-19 before | 1.02*** | 0.032*** | |  | 0.032*** | |  | 0.032*** | |  |
| Herd Immunity outlook | 1.05*** | 0.061*** | |  | 0.061*** | |  | 0.061*** | |  |

*Note:* VIF = Variance Inflation Factor; *R^2^*_global_ = auxiliary coefficient of determination  *R^2^*_auxiliary_ = global coefficient of determination; CV-19 BFS = COVID-19 Burnout Frequency Scale; FCV-19s = Fear of COVID-19 Scale. *** *p* < 0.001; ** *p* < 0.01; * *p* < 0.05. Multicollinearity was tested with VIF under a 0.1 tolerance and Klein’s rule if *R^2^*_auxiliary_ **<** *R^2^*_global_ at a 5% significance level using 10,000 bootstrap samples. ‘Employed’ and ‘Student’ demonstrated multicollinearity with ‘Age’. Analysis was conducted using the MTest package in R.^5^

# Appendix References

1. Aknin L, Andretti B, Goldszmidt R, Helliwell J, Petherick A, Neve J-E, et al. Policy stringency and mental health during the COVID-19 pandemic: a longitudinal analysis of data from 15 countries. *The Lancet Public Health* 2022; **7**.
2. Hale T, Angrist N, Goldszmidt R, Kira B, Petherick A, Phillips T, et al. A global panel database of pandemic policies (Oxford COVID-19 Government Response Tracker). *Nature Human Behaviour* 2021; **5**(4): 529–38.
3. WHO Regional Office for Europe. *A systematic approach to monitoring and analysing public health and social measures in the context of the COVID-19 pandemic: underlying methodology and application of the PHSM database and PHSM Severity Index: updated July 2022*. WHO Regional Office for Europe, 2022 (<https://www.who.int/europe/publications/i/item/WHO-EURO-2022-1610-41361-65165> [cited 11 Sep 2023])
4. Wilcox R. Chapter 12 - ANCOVA . In: Wilcox R, editor. Introduction to Robust Estimation and Hypothesis Testing (Fifth Edition). Boston: Academic Press; 2021. p. 773-780.
5. Morales-Oñate V & Morales-Oñate B. MTest: a bootstrap test for multicollinearity. Revista Politécnica 2023; **51**(2): 53-62.
